# Generating evidence on the use of Image and performance enhancing drugs in the UK: results from a scoping review and expert consultation by the Anabolic Steroid UK network

**DOI:** 10.1186/s12954-021-00550-z

**Published:** 2021-10-17

**Authors:** Jim McVeigh, Evelyn Hearne, Ian Boardley, Geoff Bates, Vivian Hope, Rob Ralphs, Marie Claire Van Hout

**Affiliations:** 1 Substance Use & Associated Behaviours, Department of Sociology, Manchester Metropolitan University, Manchester, UK; 2 Public Health Institute, Liverpool John Moores University, Liverpool, UK; 3 School of Sport, Exercise and Rehabilitation Sciences, University of Birmingham, Birmingham, UK; 4 Institute for Policy Research, University of Bath, Bath, UK; 5 Faculty of Health, Public Health Institute, Liverpool John Moores University, Liverpool, UK

**Keywords:** Image and performance enhancement drugs, IPEDS, United Kingdom, Review

## Abstract

**Background:**

The use of anabolic androgenic steroids (AAS) and associated image and performance enhancing drugs (IPEDs) is now a global phenomenon. There is a need to develop evidence to support the development of interventions to prevent the commencement of use, to minimise the potential harms or to support those in their cessation of use. While the United Kingdom (UK) is no exception to this issue, its public health and legislative response to the phenomenon differs to other countries and requires the examination of research specific to the UK. Therefore, a scoping review has been conducted to examine the recent relevant literature to help inform the development and evaluation of effective interventions to reduce the harmful use of IPEDs.

**Methods:**

A comprehensive search strategy was developed for multiple bibliographic databases, supported by and iterative citation searching process and complimented by expert input from the Anabolic Steroid UK Network. Research conducted by or UK academics or within the UK were eligible, if published in the previous five years.

**Results:**

In total 87 eligible outputs were identified, including 26 review articles, 25 qualitative papers and 24 quantitative papers. together with small numbers of clinical studies/case reports (6) and commentaries/correspondence (6). The most common topics of research were public health, treatment and harm reduction (41), followed by studies focusing on epidemiology, sub-groups of people using IPEDs and motivations for use (34). The studies illustrated the diverse populations of people who use a range of enhancement drugs including concomitant psychoactive drug use. A number of papers focused on blood borne viruses and associated issues, while others reported on the uptake of needle and syringe programmes. No effectiveness evaluations related to any aspect of treatment, harm reduction or other intervention were published during study period.

**Conclusion:**

There is a need for the development of effectiveness evaluations of current interventions and any future service provision for people using image and performance enhancing drugs. While there have been no studies of this nature to date, this review illustrates the rich data that has been gathered through diverse methodologies, that will assist in the development of future effectiveness evaluations.

**Supplementary Information:**

The online version contains supplementary material available at 10.1186/s12954-021-00550-z.

## Background

Image and performance and enhancing drugs (IPEDs) include a wide range of drugs across various pharmacological categories. Their common features are the function of their use: the alteration of physical performance, or appearance. IPEDs [1] form a subset of human enhancement drugs (HEDs) [1–3], and are predominantly those that promote lean muscle mass (e.g., anabolic androgenic steroids [AAS], human growth hormone [hGH]) but may also include weight loss products such as dinitrophenol or skin tanning injections (e.g., melanotan II). Whilst the use of IPEDs is by no means a new phenomenon, until relatively recently attention has been largely restricted to professional/elite athletes and bodybuilders. However, IPED use has moved beyond the sporting arena and is now commonplace amongst non-elite, recreational trainers within mainstream gymnasia [1–7]. This situation is not unique to the United Kingdom (UK) and other high-income countries such as the United States of America (USA), Australia and those within Western Europe. Research has identified widespread use of IPEDs in countries across the globe [8], including countries in the Middle East [9] and South America [10, 11].

The UK is unique in its response to the use of IPEDs. In the 1990s, on the recommendation of the Advisory Council for the Misuse of Drugs, a decision was made not to criminalise the personal possession of these drugs, but to focus legislation on manufacture, distribution, and possession with intent to supply [12]. Subsequently, this principle has been maintained, with adjustments to curtail purchasing of AAS from overseas websites but no change to the legality of personal possession of AAS and associated IPEDs [13]. This approach is supported by a comprehensive network of needle and syringe programmes (NSPs) across the UK. Whilst NSPs were originally established in the 1980s in response to the HIV threat posed to people who injected heroin, people who inject IPEDs now constitute the largest client group for many NSPs in the UK [13–15]. The specific situation in the UK regarding the legality of AAS possession and the engagement of large numbers of people who use AAS, with a network of NSPs provides a unique backdrop to the development of effective interventions for this population. Therefore, in order to identify relevant evidence to support the development of these effectiveness evaluations it is necessary to identify collate and review the literature that is specific to the UK.

Unlike the evidence that is specific to the AAS environment in the UK, much of our knowledge in relation to the pharmacological effects of IPEDs is generalisable from research around the world and includes an increasing body of evidence highlighting physical and psychological harms stemming from AAS use. While harms to major organs and systems, in particular the heart and cardiovascular, have long been associated with the use of AAS [16], it is only over the last decade that the significance of long-term, high-dosage AAS has become apparent [17–19]. Alongside the recognised physical and mental health impacts, new concerning evidence is emerging in relation to long-term use being associated with structural changes to the brain, deviant brain aging, and impaired cognition. Recent studies of AAS use and the brain have also concluded that AAS dependence is associated with thinner cortex in widespread regions, specifically in prefrontal areas involved in inhibitory control and emotional regulation, compared with non-dependent AAS users [20]. Recently we have also seen greater recognition of prolonged and sometimes irreversible hypogonadism in men after long-term use of AAS [21, 22] and how the symptoms of this, including reduced libido [23], may lead to continuation or resumption of AAS use. There remains significant debate regarding the issue of aggression and violence being associated with AAS use. Recent research has concluded that for some, AAS use may contribute to aggression levels [24]. Also, while there remains the need to elucidate the mechanisms involved [25], an association between aggression and AAS dependence has been identified [26].

While the majority of people who use AAS and associated IPEDs inject at least some of these drugs [27, 28],a recent review of blood borne virus (BBV) infection amongst people who use AAS and associated IPEDs highlighted the paucity of relevant robust data globally [29]. Just nine papers published since 2000 were identified, four from the UK and Australia respectively and one from the USA. Whilst several of these studies were focused on AAS use within populations of gay and bisexual men, the large-scale UK research studies recruited AAS users from NSPs or directly from gyms. The UK study of 2010/11 identified 2% of the 395 participants as HIV positive, similar levels to those seen amongst people who inject psychoactive drugs [30]. This was supported by further data collection and analyses of surveillance data dating back to 1992, using a sample of 1296 people who had injected IPEDs. Results indicated that HIV had been present within this population for some time and provided an HIV prevalence of 1% for this period [31]. As yet we do not know the route of transmission of infection and the role that sexual contact and psychoactive drug injection may play. Furthermore, we do not know if this pattern of HIV infection amongst people who inject IPEDs is mirrored in other countries. Injection site infection and injury is a widely recognised issue. Pain and inflammation at injections sites have been identified at significant levels in studies from the UK [32], USA [33], Australia [34] and Holland [35]. While poor injecting techniques contribute to these localised infections, adulterated and contaminated products, an inevitable by-product of the illicit market is an ongoing issue on a global scale and overseas [36–39].

While AAS are the most used IPEDs, polypharmacy is the norm [7, 40]. Additional anabolic substances, such as human growth hormone (hGH) and a range of new peptide hormones (e.g., growth hormone-releasing peptide-6) are commonly used. Drugs to prevent or mitigate side-effects (e.g., tamoxifen to counter gynaecomastia), human chorionic gonadotrophin (for the resumption of normal testicular function) and a range of weight loss drugs (e.g., ephedrine, dinitrophenol, clenbuterol) are also part of the established pharmacopeia. Low-cost production and distribution, combined with the increasing sourcing routes via the internet has resulted in substances that were once prohibitively expensive now being commonplace [41]. Furthermore, the use of other human enhancement drugs including melanotan II and sildenafil combined with psychoactive drugs is prevalent amongst some cohorts who use IPEDs, sometimes with significant potential for harmful interactions with certain drugs (e.g., cocaine) [42].

People who use AAS and associated IPEDs are by no means a homogenous group. Recent work has highlighted various typologies of user comprised of multiple subgroups with varied characteristics, risk behaviours and levels of engagement with support services [43–45]. While the focus of much of the research has centred on male use of these substances use, women do use IPEDs and in some cases use AAS, for those women using AAS the potential harms are more significant and sometimes compounded by an added sense of stigma and reluctance to engage with support services or healthcare [46].

The need for evidence to support our understanding of interventions that can reduce the harmful use of IPEDs is increasingly recognised (e.g. [47, 48]). As attention from the research community on IPEDs has amplified substantially in recent decades [15], the enlarged evidence base may provide valuable insights that will support those working to reduce harm amongst people who use IPEDs and ensure that approaches are based on a thorough understanding of up to date evidence. The review therefore sought to identify and explore evidence that will support the development and evaluation of effective interventions to reduce the harmful use of IPEDs. The underpinning research question developed by the research team was “how has the academic literature base on the use of IPEDs in the UK developed in the past five years and what does it tell us?”. Specifically, we sought to gain insight into the characteristics of studies investigating IPED use including: the methods used, topics of research, the characteristics of study populations, and key themes within study findings and recommendations. We conducted a scoping review of UK literature on the use of IPEDs to map and describe extant UK based literature, and in partial fulfilment of the UK National Institute for Health Research development grant (NIHR 132730), Image and Performance Enhancing Drugs (IPEDs): Assessment of available intelligence and research gaps to inform intervention evaluation’ [47]. While the international evidence base pertaining to the pharmacological effects of AAS and associated IPEDs is largely applicable to the UK, the specific situation in the UK warranted a review of the UK published literature over the last five years. It is within this environment that we sought to identify the current research landscape to ultimately inform the development of evidence based effective interventions. Therefore, this review looked specifically at the AAS/IPED outputs from UK academics/institutions. These results compliment related NIHR research activity comprising estimations of the size of the population of people who use AAS, the extent and characteristics of service provision for this group and the systems mapping of factors that influence the harmful use of IPEDs.

## Methodology

### Approach

A scoping review design was chosen to map and describe what is known about the current status and focus of research in relation to IPEDs in the UK. Scoping reviews were first proposed by Arksey and O'Malley [49] and have been further advanced by others over the last decade [50–52]. More recently, Tricco, Lillie [53] defined scoping reviews as *“a type of knowledge synthesis, follow a systematic approach to map evidence on a topic and identify main concepts, theories, sources, and knowledge gaps”*. This independent research methodology addresses broader research questions than systematic reviews can answer [49–52]. Scoping reviews are generally conducted to identify knowledge gaps, examine the extent (i.e. size), range (i.e. variety), and nature (i.e. characteristics) of a specific topic, summarise the findings of a heterogeneous body of knowledge, and propose agendas for future policy and research [49, 52, 54, 55].

### Search strategy

Arksey and O'Malley [49] five-stage iterative scoping review methodology was adhered to, namely: [1] identifying the essential research question, [2] identifying relevant studies, [3] study selection, [4] charting the data, and [5] collecting, summarising, and reporting the results. A search was undertaken in January 2021, in Liverpool John Moores University Library catalogues using the following databases: Web of Science; MEDLINE; Science Direct; PsycINFO; SPORTDiscus; CINHAL Plus; PubMed; Google Scholar, and Google. Search terms were compiled and agreed by the research team who had extensive knowledge and experience of public health, addiction, and IPED research (see Table 1).

**Table 1:** Search Terms for image & performance enhancing drug research outputs 2016–2020

*Key Word*	*Alternative*
***Image and Performance Enhancement Drugs***	“image and performance enhanc* drug*” OR “performance enhanc* drug*” OR “performance and image enhanc* drug*”
***Anabolic Androgenic Steroids***	“anabolic androgenic steroid” OR “anabolic–androgenic steroid*” OR "anabolic steroid*” OR “Formebolone” OR “Methenolone Enantate” OR “Oxymetholone” OR “Methandrostenolone” OR “Oxandrolone” OR “Stanozolol” OR “Masterolone” OR “Nandrolone” OR “Testosterone Enanthate” OR “Testosterone Propionate” OR “Testosterone Cypionate” OR “Trenbolone” OR “Boldenone Undecylenate” OR “Stanozolol” OR “Sustanon”
***Peptides***	*“human growth hormone” OR “somatropin” OR “somatrem”*
	*“melanotan” OR “bremelanotide” OR “afamelanotide”*
	“GHRP*” OR “Growth hormone-releasing peptide”
	*“Human chorionic gonadotrophin” OR “hCG”*
***Weight Loss***	*“Clenbuterol” OR “Sibutramine” OR “Rimonabant” OR “Dinitrophenol” OR “DNP”*
***Doping Control***	*“Doping” OR “anti-doping”*
***Oils, Fillers***	*“Paraffin oil injection” OR “site enhancement oil injection” OR “muscle fillers” OR “body fillers” OR “polyvitamin injection” OR “synthol injection”*
***SARMs***	*“SARMs” OR “tamoxifen” OR “raloxifene” OR “lasofoxifene” OR “bazedoxifene” OR “clomiphene citrate”*
***SERMs***	*“SERMs” OR “Ostarine” OR “Ligandrol” OR “Testolone” OR “Andarine”*
***United Kingdom***	*“United Kingdom” OR “UK” or “U.K.” OR “England” OR “Wales” OR “Scotland” OR “Great Britain”*

### Study selection

**Fig. 1:**
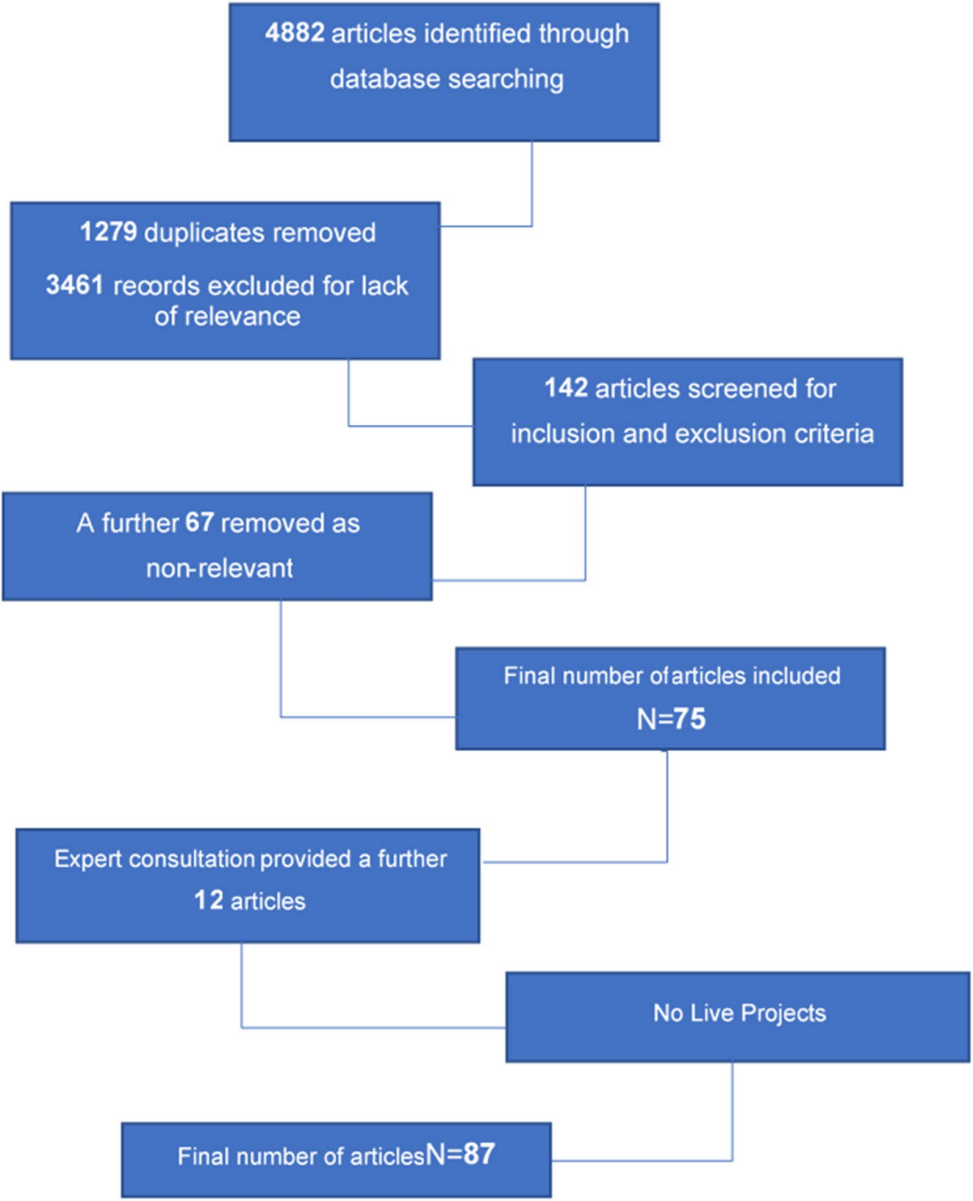
Flow chart of the search strategy used to identify image & performance enhancing drug research outputs 2016–2020

The initial search identified 4,882 articles based on the search terms outlined above (see Table 1), References were imported to Endnote® citation manager where they were organised. An initial examination of the articles indicated the possibility of many irrelevant articles. Duplicates were removed (*n* = 1279) followed by title and abstract screening of the remainder (*n* = 3461), where inclusion and exclusion criteria were applied to all citations. Studies included were: all published empirical research including articles in peer-reviewed journals and book chapters; and grey literature such as national policy reports and documents, needs assessments, service evaluations, and locally commissioned research. Date range was restricted to between January 1^st^ 2016 and December 31^st^ 2020 to capture current, relevant literature to inform the development and evaluation of effective interventions to reduce harmful IPED use, and studies conducted by UK academics or those with a UK focus due to the unique situation in the UK relating to legislation and NSP provision. Academic theses, animal models, and in-vitro studies were excluded. A total of 77 records were identified at this stage of the search (see Fig. 1). Papers were subsequently reviewed and screened to ensure those included met the inclusion criteria and discrepancies resolved [54]. Manual searching of the reference lists of the 77 records was conducted to identify any relevant literature that was not captured in the initial search. Subsequently, consultation with academics and healthcare professionals with relevant expertise (accessed through the Academic Steroid UK Network) was conducted to ensure all relevant literature was included, as recommended by Daudt, van Mossel [54]. This was a valuable step in the process as the depth and breadth of knowledge each expert brought strengthened the review and consequently, the richness of the findings. A final number of 87 sources were included in the review (see Fig. 1).

### Data extraction and charting

Data were extracted to form a dataset which included all author names and institutions, year of publication, aim and method, population, key findings, implications for policy and practice, and identified research gaps in each source.

## Results

Additional file 1: Table 2 provides a detailed overview of UK academic publications on the use of anabolic androgenic steroids and IPEDs, presenting an overview of this research to illustrate its volume and main characteristics, together with summaries of key findings.

### Profile of studies reviewed

**Fig. 2:**
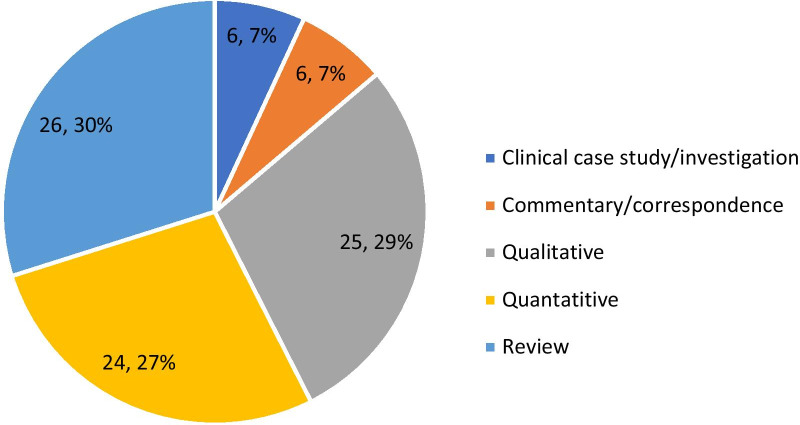
Methodologies of image & performance enhancing drug research outputs 2016–2020

The final sample of 87 records present a range of methodologies and foci on AAS and other IPEDs within the UK. The majority of evidence was gleaned from the review of 69 journal articles and supplemented by 12 relevant academic book chapters and six public health reports. There is no discernible trend in the volume, methodologies used or focus of research outputs over the five-year period. Apart from 2019, the number of outputs per year ranged between 12 and 17 publications. The high number of outputs in 2019 [29] can be explained by the inclusion of 8 chapters from one book on the use of human enhancement drugs [1]. These records are comprised of a variety of methodologies and approaches including qualitative, quantitative and review. In a small minority of cases several methods were reported within the research output, however in all cases there was a clear predominant method applied. For example, the book chapter ‘The supply of image and performance enhancing drugs (IPED) to local non-elite users in England [56], forensic analysis of a small number IPEDs was used to support the findings of two qualitative studies and was therefore recorded as a qualitative paper. Figure 2 illustrates these methodologies in an aggregated form. The 26 review articles (including systematic, scoping, and non-specific reviews) account for the largest proportion of outputs(30%), followed by 25 predominantly qualitative (29%), 24 quantitative papers (27%) with 6 (7%) commentary papers and clinical case studies respectively.

**Fig. 3:**
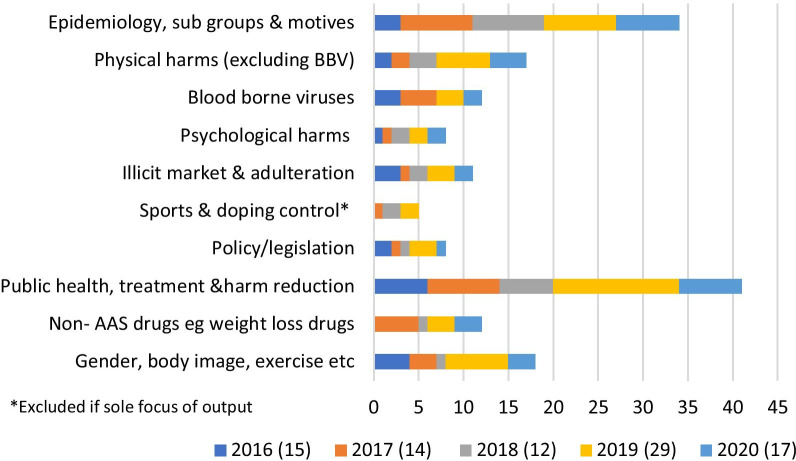
Focus of image & performance enhancing drugs research outputs 2016–2020

Additional file 1: Table 2 also includes the main topics of each output. It is important to note that while the category of sport/doping control is included, outputs were excluded if this was the sole area of focus. Outputs may be attributed to two are more categories. Figure 3 summarises these categories within the year of publication. The most common topic of research, included in 41 academic outputs, was public health/care (including harm reduction and treatment). This was consistent across each year apart from 2018 when epidemiology was the major category, this being the second most common topic overall [34].

### Key findings

The UK research from the last 5-years provided a broad spectrum of evidence related to various facets of IPED use, from across the full extent of the UK. An indication of the diversity of drugs used is provided, together with specific implications. The vast majority of the work was in relation to the use of AAS includingone paper looking at the concomitant use of stimulants [42] Four papers focusing solely on the use of weight loss drugs [57–60], two papers examining the use of the skin tanning drug melanotan [61, 62], two academic outputs describing the emerging use of the respective peptide hormones metformin and CJC-1295 [63, 64], and one article examining the use of synthol (a site enhancement oil) [65]. One paper focused specifically onthose solely taking oral AAS and associated drugs [66], two papers examined the relationshipbetween supplements IPED use [67, 68], whilethe role of concomitant psychoactive drugs was emphasised as a cause for concern in one paper [7]. Six outputs provided accounts of the ease of availability and affordability of drugs within a dynamic IPED market [56, 69–73], with the internet playing an important role with a significant market crossover with other drug using populations [74–77].

Research focused on epidemiology, sub-groups, and motives, indicates a complex landscape of diverse sub-populations of IPED users, going beyond the stereotypical young male AAS user. Different populations and practices were identified with specific practices and risks. Significant sub-groups included women using a range of substances [78–82] and older men using AAS [28]. While research that only focused on elite sport and doping control was excluded, sport clearly plays a significant role in the use of IPEDs [83] and remains a public policy concern [84]. IPED use was also associated with specific occupations as diverse as those in the military to those engaged in dance [85, 86]. The majority of the literature in this review examined the use of AAS, in particular the use of AAS by men. Only four papers solely focused on the use of IPEDs by women [57, 64, 78, 81].

This literature also provides an improved understanding of some of the motives for commencement of use or abstinence, in particular those related to self-esteem, body image and masculinity [87–96]. The continuation or resumption of AAS use driven by symptoms of hypogonadism [97–100] was a significant finding, together with wider issues related to sexual health [101]. Harms associated with IPED use were commonly described [18, 19, 102, 103], together with the risk environment [6] and efforts by individuals to mitigate these adverse consequences [104].

Common features within studies centred on public health, treatment, and harm reduction included the increasing number of AAS users engaging with NSPs [28, 105, 106] and barriers to service engagement including a lack of confidence in practitioners’ knowledge, perceived stigma. A failure to recognise the beneficial effects of AAS was identified together with an overemphasis of the harms [107–110], while the need for non-judgemental specialised services was emphasised [111–119]. A greater understanding of the need for a multi-layered approach to preventing both IPED use in general, and harmful use in particular, was emphasised [91, 120, 121]. The need for an understanding of the culture as well as the behaviours of people who use IPEDs was deemed essential, together with an appreciation that IPEDs may have potential benefits to the user [58, 108, 110].

Another recurring theme within this literature was the need for health practitioners to demonstrate both a non-judgemental attitude and have a demonstrable level of knowledge of both IPEDs and how they are used. This was considered a prerequisite if the current barriers to service engagement are to be overcome [110]. Practitioners must have an appreciation of the complex relationship between AAS use and body image [96] and an understanding that there is a perceived normalisation of IPED use within some social groups, with concepts around masculinity and muscularity being highly influential on decisions to use IPEDs [91]. While the majority of the literature referred to practitioners in general, the potential for an increased role for both social workers [115] and endocrinologists [116] was highlighted.

Various aspects of a potential continuum of care and support were also discussed. These ranged from prevention activities within a generic health promotion approach, recognising the complex factors that make children, youth and adults vulnerable to IPEDs [42, 120, 121], through to the need for relapse prevention to divert former AAS users from a focus on their body as their major source of self-esteem [94]. However, the main area of discussion related to existing and potential harm reduction interventions and services [7, 15, 111]. In addition to the expansion of innovative development and activity within NSPs [107, 122], other venues and modes of engagement to promote sexual health [101] and ensure non-stigmatising environments were called for [28, 29, 123, 124]. There were also calls for the implementation of effective assertive outreach [125] and the adjustment of health and social care settings to enhance on-site engagement with people who use IPEDs [107].

As part of service provision there were also calls for comprehensive testing including physiological indicators of harm [112] together with testing for BBVs [126] and psychosocial support regarding body image. Such testing for BBVs could draw upon the improved understanding of injecting beliefs and behaviours [101], including BBV risks [25, 27, 102–107], evident in the review. Information of nutrition and exercise [113], building self-esteem [97], psychological services to address dependence and muscle dysmorphia [82, 97], and other body image vulnerabilities [59] were also considered necessary. There were consistent findings regarding the need for practitioners to have an understanding of the diverse populations of people who use IPEDS [45], the episodic nature of use [74], polydrug use [7], those who do not inject [66] and those using drugs other than AAS [58, 60, 127]. An understanding of the market was considered necessary in order to provide credible health related information on risks of active ingredients and the quality products obtained through the illicit market [64].

## Discussion

The diversity of UK published research between 2016 and 2020 reflects a growing scientific and academic interest in this phenomenon and underpins the complex issues related to the use of available IPEDs. They also indicate the multidisciplinary response that is required if, as researchers, practitioners, policy makers, and all importantly, people who use these drugs, are going to synergise and work collaboratively to raise awareness increase our understanding and ultimately reduce the harms associated with use. The research findings, together with the stated implications for policymakers, practitioners and the research community illustrate the broad spectrum of opportunities to reduce harm, including prevention, diversion, treatment, cessation support and policies that directly affect the illicit market. However, notable by their absence and as highlighted within many of the publications is the lack of robust effectiveness of evaluations in relation to interventions focused on the use of IPEDs. While the focus of this paper is on recently published UK research, due to the unique position we are in, in relation to legislation and needle and syringe provision, it is worth noting that this dearth of intervention effectiveness evidence is replicated around the globe.

Findings also underscore the need for future efforts to develop and evaluate interventions should see the involvement of people who use IPEDs as an essential component during all stages of the research process. For example, many included records emphasised that interventions should encompass peer support groups and educators [113, 125], and a client-centred approach [79, 106]. Thus, there was a recurring theme within the UK literature of the recognition of the need to effectively engage with the communities of people who use IPEDs. It is a widely held belief that the required interventions and the research that is needed to test and evaluate them can only be achieved with the participation of those who use IPEDs. This belief is supported not only by the IPED-specific literature reviewed here, but also by the broader literature relevant to the development and evaluation of interventions aimed at enhancing or protecting physical and psychological health. Often referred to as patient and public involvement (PPI), research funders now frequently endorse and sometimes obligate PPI during all stages of health and social care research [129, 130]. Incorporating PPI can benefit research by promoting recruitment [131], which can enhance its validity and reduce costs [132]. Moreover, the efficacy of PPI is greatest when those with lived experience of the behaviour/condition being studied are represented as research partners, supporting the benefits of harnessing knowledge from such experience-based experts alongside that of scientists and professionals when designing, delivering, and disseminating research [133]. The importance of involving those with lived experience of IPED use in research and in developing and evaluating harm reduction interventions for IPED use is perhaps even more important than in other fields given evidence demonstrating a lack of trust in healthcare professionals when it comes to IPED use [134–136].

The research literature provided a wealth of far-reaching recommendations for future research. It is essential that researchers engage and collaborate with the communities of people who use IPEDs to better understand the patterns of drug-use behaviours, motives and associated risks [28]. Future research should focus on delivery of holistic healthcare and early intervention for those attending NSPs and outreach services [42]. However, harm reduction should look beyond NSPs to meet the needs of those who do not inject [66].

While the evidence base related to harms stemming from AAS use has developed over the last 5 years, there needs to be a continued focus on specific adverse effects for the full range of IPEDs [18, 19, 65, 117, 123], so that policy makers have a comprehensive understanding of the drivers and motives for use and cessation, and the associated harms for AAS [6, 79, 91, 114, 117, 124] and other IPEDs [15, 57–61, 64, 68, 73, 104, 122, 128]. We should also develop the evidence and increase our understanding of the issues associated with the diverse populations that use IPEDs [95, 105]. More specifically, research should focus on those people using IPEDs who may be particularly vulnerable [76], including women [78, 81], those in prison [117], those with specific occupations and those engaging in pertinent activities such as sport [67, 83, 85, 86, 90, 93].

Motives for commencement, continuation, and potential cessation of IPED use should also receive continued research attention [92, 98, 120]. Key examples identified for further attention include body image [96, 97, 129], muscle dysmorphia [94], muscularity [82], masculinity [87, 130], and hypogonadism [99, 113, 115]. Research findings indicate a combination of routinely available data, survey data and other novel data collection methods should be employed [95], including online methodologies to gain a better understanding of the prevalence of use and associated behaviours [15, 45, 64, 74, 116, 127]. Research is also needed into the clinical treatment of adverse effects [102] and how stigma and barriers within generic health services may be effectively addressed [112]. This is especially pertinent in relation BBV services [28, 31, 119, 126, 131, 132] and sexual health services [31], including men who have sex with men and women who have sex with women [101]. We also need to gain a better understanding of the supply and distribution of IPED, the role of the internet, potential for a more regulated market and the harms caused by the illicit market [6, 7, 15, 63, 69, 73–75]. The overarching them in relation to identified research gaps and recommended focus of attention is the evaluation of interventions to identify cost-effective demand reduction and harm reduction solutions [104, 105, 107, 111, 128]. Further attention is warranted by policymakers and public health surveillance systems to track and monitor this emerging and increasingly mainstream form of body modification and consumerism of IPED pharmaceuticals.

## Conclusion

The UK IPED research community has been highly active over the last five years. This review evidences a wealth of data relating to people who use IPEDs, including their practices and associated harms. However, where interventions are a focus of the research, findings tend to be descriptive and their remains a paucity of effectiveness studies. The literature highlights the need for meaningful involvement of people with lived experience, reinforcing the principles of co-production in the development of future intervention evaluations to reduce the harms associated with this form of substance use.

## Supplementary Information

**Additional file 1: Table 2**. Charted Records of United Kingdom publications on anabolic androgenic steroids and associated IPED use (2016-2020).
