# Supplementary material for: Generating evidence on the use of Image and performance enhancing drugs in the UK: results from a scoping review and expert consultation by the Anabolic Steroid UK network
Source: Harm Reduct J. 2021 Oct 17;18:107. doi: 10.1186/s12954-021-00550-z (PMC8522223; doi:10.1186/s12954-021-00550-z)
Supplement: Supplementary file 1 — Additional file 1: Table 2. Charted Records of United Kingdom publications on anabolic androgenic steroids and associated IPED use (2016-2020). [file 12954_2021_550_MOESM1_ESM.docx]

**Table 2 Charted Records of United Kingdom publications on anabolic androgenic steroids and associated IPED use (2016-2020)**

| **First Author; Year; Title.**  **Institution of first author and location.** | **All Authors** | **Aim and Method** | **Population** | **Key Findings** | **Implications for Policy** | **Implications for Practice** | **Identified Research Gaps** | **Main topic or focus** |
| --- | --- | --- | --- | --- | --- | --- | --- | --- |
| Zahnow, R.  2020  [**Motives and Correlates of Anabolic-Androgenic Steroid Use with Stimulant Polypharmacy.**](https://doi.org/10.1177%2F0091450920919456)  School of Social Science, The University of Queensland, St Lucia, Brisbane, Queensland, 4072, Australia.  **Co-authors: UK** | McVeigh, J.  Bates, G.  Winstock, A. | **AIM:** To explore polydrug use in male AAS users.  To examine the association between motivations for AAS use concurrent with stimulant use.  **METHOD:** Retrospective data analysis of the Global Drug Survey 2015. | General population. | - Those who use AAS for aesthetic purposes are more likely to report concurrent use of AAS and stimulants than those who are not motivated by weight loss goals. - Individuals reporting concurrent use of stimulants and AAS were more likely to engage in heavy drinking. - Findings highlight the need for continued reviewing and renewal of drug safety messages and a move away from the assumption of male athlete AAS use. | Steroid prevention efforts should be targeted at the general population and to include education, and health promotion strategies.  Drug education at schools should include AAS to their programs. | Steroid prevention efforts should be targeted at the general population and to include education, and health promotion strategies. | Future research should focus on delivery of holistic healthcare and early intervention for those attending NSPs and outreach services. | **⚫**  **⚫**  **⚫** |
| Whyte, I.  2020  [**Performance and image enhancing drugs use in active military personnel and veterans: A contemporary review.**](ttps://doi.org/10.1002/tsm2.186)  Faculty of Health Sciences and Wellbeing,  University of Sunderland, Sunderland, UK | Pattinson, E.  Leyland, S.  Soos, I.  Ling, J. | **AIM:** to scrutinize  the literature on the prevalence and motivations for IPED use.  among serving military personnel and veterans.  **METHOD:** Review | Service personnel: The Army,  Air force, Navy, Marines, & Veterans. | - AAS and weight-loss IPEDs most commonly used. - Primary reason for AAS use was for image enhancement. - Second reasons for IPED use were to have the ability to work through physical and emotional demands of active duty. - Those most likely to use IPEDs were young non-commissioned Army personnel. | A policy shift to address an “acceptable” culture of IPED use in Army personnel is warranted. | Better support for Army personnel IPED users on transitioning out of the military service is needed.  This will impact health services such as the NHS by reducing complications from IPED use. | Further research is required to explore active military personnel’s reason for IPED use and how they access them.  Research should also investigate and understand IPED use initiation, particularly whether it is pre-, during, or post-military service. | **⚫**  **⚫** |
| Turnock, L.A.  2020  [**Inside a steroid ‘brewing’ and supply operation in South-West England: An ‘ethnographic narrative case study’.**](https://doi.org/10.1016/j.peh.2019.100152)  Department of Applied Criminology and Forensic Studies, University of Winchester, Winchester, United Kingdom. |  | **AIM: To** explore UK IPED markets to understand how both ‘homebrewing’ and local market  steroid supply operates.  **METHOD:** Ethnographic narrative case study. | Competitive powerlifter  who became a steroid importer, ‘brewer’ and supplier. | - The availability and accessibility via the internet of ‘raw’ powder supplies from China has resulted in changes to the market as AAS do not require processing in intermediate countries for Western consumption. - The study highlights how homebrewing may impact quality of end product at the gym-level. - Study found that different steroid brands have come from the same lab but is labelled by gym-level suppliers. | Specific research exploring IPED manufacture and supply will likely impact policy in the future. | Not discussed. | Future research should explore these findings further in relation to above gym-level supply and distribution of this market. | **⚫** |
| Mullen, C.  2020  [**Anabolic Androgenic Steroid Abuse in the United Kingdom: An Update.**](https://doi.org/10.1111/bph.14995)  School of Computing, Engineering and  Physical Sciences, University of the West of  Scotland, Paisley, UK. | Whalley, B. J.  Schifano, F.  Baker, J. S. | **AIM:** Review current AAS use/abuse in the UK.  **METHOD:** Review | - | - AAS users predominantly late 20s-early 30s, heterosexual males, recreational exercisers, weight training. - Most UK AAS users initiate use older than 18 years. - Health supplement consumption is associated with AAS use. | Improved regulation of the health supplement market is warranted as some have been found to contain AAS.  Further research will guide policymakers and public health initiatives to target those most at risk for AAS use. | Interventions targeted at improved sexual behaviours needs to be considered.  Individuals within the AAS using community should be better represented particularly adolescent boys and females. | Research investigating negative health effects of AAS use, specifically on cardiovascular system, cerebrovascular, renal, and hepatic systems,  and cognitive function. | **⚫**  **⚫**  **⚫** |
| McVeigh, J.  2020  [**A sentinel population: The public health benefits of monitoring enhanced body builders.**](https://doi.org/10.1016/j.drugpo.2020.102890)  Substance Use and Associated Behaviours, Department of Sociology, Manchester Metropolitan University. | Salinas, M.  Ralphs, R. | **AIM:** To review PH surveillance of AAS users specifically innovators in their community.  **METHOD:** Commentary | Enhanced bodybuilders. | - Enhanced bodybuilders are a sentinel population that should be observed. - Effective monitoring and early identification of AAS diffusion will support effective interventions and reduce harms. - Engagement and communication with HED “innovators” and “early adopters” to overcome barriers and mistrust is essential for collaboration | Development of an early warning system to monitor enhanced bodybuilders to identify potential diffusion of drug use behaviours to the wider population. | The need to effectively engage with enhanced bodybuilder to understand practices and develop prevention, harm reduction and cessation support. | Influential enhanced bodybuilders’ trends and practices experiences, and the diffusion of these to  the wider AAS using communities should be monitored and collated. | **⚫**  **⚫**  **⚫**  **⚫** |
| McCullough, D.  2020  [**How the love of muscle can break a heart: Impact of anabolic androgenic steroids on skeletal muscle hypertrophy, metabolic and cardiovascular health.**](https://link.springer.com/article/10.1007/s11154-020-09616-y)  Research Institute of Sport and Exercise Science, Liverpool John  Moores University, Liverpool, UK. | Webb, R.  Enright, K. J.  Lane, K. E.  McVeigh, J.  Stewart, C. E.  Davies, I. G. | **AIM:** To highlight the mechanisms by which AAS exert their hypertrophic effects on skeletal muscle; to explore the impact of AAS use on lipid, lipoprotein, and glucose metabolism; and to explore the negative effects of AAS withdrawal and potential treatments.  **METHOD:** Review | - | - Chronic AAS use effects metabolism thus increasing the risk of CVD. - Growing prevalence of AAS use will intensify current rates of CVD. - Chronic AAS use also results in metabolic syndrome and associated dysregulated metabolic health. - These health issues are more generally associated with the sedentary/obese population. | Not Discussed | Educational and psychological interventions can effectively manage AAS and AAS-related polypharmacy, and guidance on cessation of use. | Future research is required on long-term AAS use effects on markers of metabolic health. This will provide precise information of potential harms of AAS use in males and females.  Treatments to support AAS cessation and combat adverse metabolic health outcomes of AAS users. | **⚫** |
| Kotze, J.  2020  [**‘Looking ‘acceptably’ feminine: A single case study of a female bodybuilder’s use of steroids’.**](https://doi.org/10.1016/j.peh.2020.100174)  Teesside University, UK | Richardson, A.  Antonopoulos, G. A | **AIM:** To highlight the motivations for and methods of female AAS use; to explore the connections between the competitive logic of liberal-postmodern consumer capitalism, ‘competitive femininity’ and steroid use.  **METHOD:** In-depth ethnographic interview | Female bodybuilder | - The female bodybuilder is hyper-conforming to cultural norms through the over-identification of a hyper-idealised form of what constitutes ‘acceptable femininity’. - The consumption of AAS is strongly associated with the desire for aesthetic appeal. - Both long- and short-term motivations for AAS use are grounded in a drive for conformity. | Not Discussed. | Implications for health practitioners for reducing AAS consumption by advising users of possible adverse effects on their health. | Future research to gain an understanding of motivations for females to use AAS and what their preferred method of consumption is, which will aid in harm reduction and mitigate negative effects. | **⚫**  **⚫** |
| Hope, V.  2020  [**Factors associated with hepatitis C and HIV testing uptake among men who inject image and performance enhancing drugs.**](https://doi.org/10.1111/dar.13198)  Public Health Institute, Liverpool John Moores University, Liverpool, UK. | McVeigh, J.  Begley, E.  Glass, R.  Edmundson, C.  Heinsbroek, E.  Kean, J.  Campbell, J.  Whitfield, M.  Morgan, G.  Acreman, D.  Smith, J. | **AIM:** To examine  the uptake of HCV and HIV diagnostic testing among IPED injectors in the UK.  **METHOD:** Retrospective Data Analysis of the IPED Survey 2016. | Males who inject IPEDs (n=562) recruited through community and health services. | - 2/3rds of IPED injectors had never been tested for HCV or HIV. - Uptake of testing associated with psychoactive IDU, history of imprisonment, and sexual risk. - Uptake also associated with recent metabolic or physiological testing pertaining to AAS use. - Uptake or offer of testing not reported by 50% of participants, who had been to a health service. | Not discussed | It is recommended that healthcare services offer BBV testing along with metabolic and physiological tests, which may increase uptake.  Dedicated specialist IPED clinics and IPED workers at NSPs in line with national guidelines is warranted. | Future research should focus on barriers to uptake of BBV testing among IPED users in primary care settings.  Research is needed to inform the development of interventions and best practice for targeting interventions. | **⚫** |
| Hope, V.  2020  [**Facilitators and barriers to health care access amongst people using image and performance enhancing drugs in Wales: Findings & Outcomes Report.**](https://phw.nhs.wales/publications/publications1/facilitators-and-barriers-to-health-care-access-amongst-people-using-ipeds-in-wales-english/)  Public Health Institute, Liverpool John Moores University, Liverpool, UK. | Leavey, C.  Morgan, G.  Acreman, D.  Turner, D.  Smith, J. | **AIM:** To verify  barriers and facilitators people injecting AAS and other IPEDs face when accessing health services.  **METHOD:** Qual Interviews with AAS users, community-based HCPs, and an Evidence Gathering and Synthesis | AAS users and HCPs | - AAS users felt motivating factors for use were undervalued and misunderstood by HCPs. - Majority of AAS users minimised the health risks even though they experienced adverse effects. - All participants concerned re lack of evidence-based information for both AAS users and HCPs. - Most AAS users reluctant to disclose use to HCPs or seek healthcare due to fear of judgement/stigma. - Growing use of online methods for obtaining injecting paraphernalia and accessing metabolic testing by AAS users as it was deemed more accessible. | It is recommenced that public health bodies, community substance misuse leads, and commissioners develop best practice guidance for the implementation of effective assertive outreach facilities, and the adjustment of health and social care settings to enhance on-site engagement. | Public health and professional bodies should develop AAS training for healthcare professionals and provide knowledge sharing opportunities.  Substance misuse commissioning boards are advised to adopt a whole person approach to undertaking evaluation of local health and social care services available to people who use AAS. | Research resources should be provided by academic institutions, government bodies, and health services to undertake an evaluation for the development of the evidence-based on online technologies that aid healthcare service engagement.  Collaboration between public health bodies and UK wide academic institutions is recommended to undertake research to ascertain.  prevalence of AAS use and evidence of harms associated with use. | **⚫** |
| Hibbert, M.  2020  [**Image and performance enhancing drug use among men who have sex with men and women who have sex with women in the UK.**](https://doi.org/10.1016/j.drugpo.2020.102933)  Public Health Institute, Liverpool John Moores University, Liverpool, UK. | Brett, C. E.  Porcellato, L. A.  Hope, V. D. | **AIM:** To examine the scope of IPED use among  MSM and WSW in the UK; and to investigate the factors associated with their use.  **METHOD:** Cross-sectional survey | MSM (*n=*1,663) & WSW (*n=*1,513). | - 1 in 25 MSM & WSW used IPEDs in the previous year. - Recent IPED use associated with psychoactive drug use and STI diagnoses. - Both MSM & WSW reported body dissatisfaction and lower satisfaction with life as reasons for IPED use. - MSM IPED use was associated with lower sexual self-efficacy. - IPED use among MSM was associated with drugs commonly related to chemsex in the UK. - WSW who use IPEDs were more likely to have attended a GUM clinic than non-IPED using WSW indicating a significant area for IPED HR provision for WSW. | Not Discussed | GUM clinic significant area for IPED harm reduction provision for WSW.  Psychosocial support for body dissatisfaction and satisfaction with life is recommended.  Specialist psychological support for IPED relates issues such as historical sexual assault or internalised homophobia. | Future research is needed to explore the associations between IPED use and body dissatisfaction and satisfaction.  with life among MSM & WSW.  An investigation is required to confirm if there is a relationship between sexual satisfaction, sexual risk.  behaviours, and the use of erectile dysfunction drugs.  Demographic variations pertaining to IPED use among WSW is warranted. | **⚫**  **⚫** |
| Henning, A.  2020  [Preventing, producing, or reducing harm? Fitness doping risk and enabling environments.](https://doi.org/10.1080/09687637.2020.1865273)  Faculty of Health Sciences and Sport, University of Stirling, UK | Andreasson, J. | **AIM:** To compare two distinct approaches to fitness doping: Sweden’s prevention-based approach and Scotland’s harm reduction approach.  **METHOD:** Comparative case study analysis |  | - HR efforts are reduced and risks are increased for individuals who use these substances due to restrictive anti-doping policies. - Less restrictions may promote more HR work but social, economic, and policy risks may remain. - People who use IPEDs in both the UK and Sweden respond to prohibitive measures by going to an online environment where discussions are centred on HR and enabling IPED use. | A policy shift away from policing and punishment to a more accepting and supportive focus is recommend.  Policymakers should consider Scotland’s Glasgow IPED clinic which offers a wide range of services such as bloodwork, substance testing, and information regarding safe use. | Harm reduction services with a wide range of services for IPED users is encouraged. | Future research to assess the effectiveness of HR strategies as well as the impact of online communities in promoting HR for IPED users, specifically AAS. | **⚫**  **⚫** |
| Harvey, O.  2020  [**Support for non-prescribed anabolic androgenic**](https://doi.org/10.1080/09687637.2019.1705763)  [**steroids users: a qualitative exploration of their**](https://doi.org/10.1080/09687637.2019.1705763)  [**needs.**](https://doi.org/10.1080/09687637.2019.1705763)  Faculty of Health and Social Sciences, Bournemouth University, Bournemouth, UK. | Parrish, M.  van Teijlingen, E.  Trenoweth, S. | **AIM:** To ascertain  the types of support needed by people who use non-prescribed AAS.  **METHOD:** Qualitative Interviews | Male AAS users | - AAS users expressed a desire to consult with medical professionals regarding their use; to see more evidence-based research; and were willing to pay for healthcare services. - Less than half of the participants in the UK had accessed NSPs. - Participants felt that focusing on cessation of use was unrealistic and unhelpful, and that medical support was warranted. - Participants wished for professionals to have personal experience of AAS use. | Governments should consider increasing evidence of a more personalised approach to AAS user groups and from academic communities.  Public health initiatives are encouraged to establish specialist steroid services. | Peer support groups are encouraged to address the expressed wishes of participants for HCPs to have AAS use experience.  Access to nutrition and exercise advice at traditional services is recommended to prevent initiation to use at a younger age.  Testing for substances quality is warranted.  Delivery of services should be well-trained, knowledgeable, and non-judgemental. | Participants suggested a need for more human-based scientific studies to be carried out.  Further research is needed to explore the impact of low testosterone on quality of life among AAS users.  A better understanding of motivations for use is needed and will possibly impact which supports are needed across the life-course of the individual. | **⚫** |
| Greenway, C.  2020  [**Muscle dysmorphia and self-esteem in former and current users of anabolic-androgenic steroids.**](https://doi.org/10.1016/j.peh.2019.100154)  School of Psychology, University of Wales Trinity Saint David, West Wales, UK. | Price, C. | **AIM:** To examine the extent of low self-esteem in men in the weightlifting community; to compare levels of MD symptomology and self-esteem between current, former, and non-users; and to explore the motivations behind initial use, continued use and cessation, and whether MD is a precursor or cause of AAS use.  **METHOD:** Demographic questionnaire; MASS scale; RSES scale; and Open-ended questions. | Non-competitive weightlifters; current users from a needle exchange programme (NEP); gym users in the UK. | - Low self-esteem identified as a risk factor for AAS use but Improved self-esteem was a motivator for continued use. - Image enhancement rather than performance enhancement was the primary motivation for use. - Improved physique was considered a motivation for cessation as there was no longer a need for AAS. - Settling down with a partner was another motive for cessation. | DSM-IV needs to consider the lack of criteria for AAS dependency based on current evidence.  The development of prevention measures that will decrease muscle dysmorphia and AAS use. | Harm reduction services and engagement with this vulnerable community with specific focus on building self-esteem is needed.  Significant need for psychological services to address dependency because of secondary reinforces. | Future research is required to examine the role of body image and MD on dependence of AAS.  Exploration of former AAS users body image and self-esteem are warranted and will inform future interventions that support abstinence.  Research is needed to examine life-histories of these men and establish the precursors to the development of MD. | **⚫** |
| Gilmore, H.  2020  [**Help-Seeking Beliefs Among Anabolic Androgenic Steroid Users Experiencing Side Effects: An Interpretive Phenomenological Analysis.**](https://doi.org/10.1123/jcsp.2019-0028)  Sport and Exercise Sciences Research Institute, Ulster  University, Jordanstown Campus, Newtownabbey, Northern Ireland. | Shannon, S.  Leavey, G.  Dempster, M.  Gallagher, S.  Breslin, G. | **AIM:** To determine AAS users’ experience of side effects and help seeking  Beliefs.  **METHOD:** Interpretative Phenomenological Analysis of six interviews | AAS Using recreational fitness clients and non-elite athletes.  Males *n=*6 | - Motivations for AAS use were for cosmetic reasons, to gain size sport, and self-medication for self-diagnosis of low testosterone. - Individualised methods for cognitive dissonance, risk-benefit analysis, and underground self-treatment occurred because of reluctance to seek professional care. - Four main themes: a belief that GPs and medical staff are ineffective; biasing harms and benefits; a subculture in facilitating and sharing information; maladaptive harm minimization. | The development of educational interventions for athletes and fitness people is needed. | Clinic healthcare providers and practitioners are advised to adopt a client-centred approach when communicating awareness, educating, and counselling AAS users. | Research is warranted to explore possibilities for future therapeutic approaches for athletes from perspectives of all stakeholders. | **⚫**  **⚫**  **⚫** |
| Germain, J.  2020  [**2,4 dinitrophenol: It's not just for men.**](https://doi.org/10.1016/j.drugpo.2020.102987)  Public Health Institute, Liverpool John Moores University, Liverpool, UK. | Leavey, C.  Van Hout, M.C.  McVeigh, J. | **AIM:** To provide an overview of female experiences of DNP use.  **METHOD:** Online research method. Thematic analysis of discussion threads; semi-structured interviews. | 440 discussion threads pertaining to female DNP use. 4 male and 1 female interview. | - DNP use had the desired effects including increased energy, weight loss, suppressed appetite and increased confidence because of weight loss. - Side effects most reported were overheating, sweating, diarrhoea, constipation, hives, and an irregular menstrual cycle. - Weight loss results outweighed the adverse effects even though many experienced anxiety over these effects. - Many women searched for advice from online forums. - Females trusted the information on the forum even though it is mainly aimed at males and women often faced disapproval and stigma. - DNP use among women was considered novice use, whilst men were seen as experts | DNP use needs to be considered as part of a wider health agenda. | GPs and other healthcare professionals should be educated and knowledgeable on the harmful effects of DNP and the ease of access via the internet to weight loss products. | Not Discussed | **⚫** |
| Cranswick, I.  2020  [**Oh take some man-up pills”: A life-history study of muscles, masculinity, and the threat of injury.**](https://doi.org/10.1016/j.peh.2020.100176)  Research Institute of Sport and Exercise Sciences, Liverpool John Moores University. | Richardson, D.  Littlewood, M.  Tod, D. | **AIM:** To understand the role muscularity plays in the masculine identities of weight-training males.  **METHOD:** Life-history semi-structured, interviews | Males, recruited via an injury clinic, who reported that injury had threatened their masculine identities. | - The role of muscularity was central to a form of aesthetic and bodily capital. - Men created different realignment narratives to restore and sustain their masculine identity in times of threat. - Social observations and interactions influenced narrative and identity constructions and the significance attached to muscularity. | Rehabilitative professionals need to consider masculine narratives, and construct injury experiences that support the person’s narratives, values, and attitudes. | Not Discussed | Future research should focus on encompassing diversity in, and socially dependent nature of muscular masculinity of men. | **⚫** |
| Amaral, J.M.X.  2020  [**Effective treatment and prevention of attempted suicide, anxiety, and aggressiveness with fluoxetine, despite proven use of androgenic anabolic steroids**](https://doi.org/10.1002/dta.2912)**.**  King's College London Institute of Psychiatry, Psychology and Neurosciences (IoPPN), Addictions Department, London, UK. | Padilha, M. C.  Chagas, S. V.  Baker, J. S.  Mullen, C.  Vieira Neto, L.  Aquino Neto, F. R.  Cruz, M. S. | **METHOD:** Clinical Case Report | 24-year-old male, 6-year history of AAS use. | - Anxiety, aggression, depressive symptoms, and a suicide attempt resulted in a male following withdrawal symptoms of AAS use. - 30 days of inpatient treatment followed the suicide attempt. - Fluoxetine alleviated the symptoms of post-cycle depression, prevented further aggression, anxiety, and suicidal ideation despite subsequent use of AAS. | Not Discussed | The risk of drug interactions between AAS and Fluoxetine should be closely monitored given the risk for mania by both substances. | Future controlled studies are recommended to explore the mechanisms of fluoxetine effects over AAS-related psychiatric symptoms. | **⚫**  **⚫** |
| Woodward, C.  2019  [**Hepatocellular carcinoma in body builders; an emerging rare but serious complication of androgenic anabolic steroid use.**](https://dx.doi.org/10.14701%2Fahbps.2019.23.2.174)  University Hospital Wales, and Public Health Wales, 19 Nant-Y-Wedal, Cardiff CF143QU, UK. | Smith, J.  Acreman, D.  Kumar, N. | **METHOD:** Clinical Case Report | 39-year-old male, bodybuilder.  28-year-old male, bodybuilder. | Case 1:  Presented with abdominal pain. Ultrasound revealed a liver lesion. Ceased AAS use 18 months prior to this. MRI showed a hepatocellular carcinoma. vascular tumour removed surgically. 3month review showed no recurrence.  Case 2:  Presented with epigastric pain.  Several liver lesions with bleeding into the lesion  in the left liver were observed following imaging. >5-year history of AAS use. Cardiomyopathy 5 years ago attributed to AAS use. Surgical removal of mass on liver. 3-month review showed no recurrence which coincided with cessation of AAS use. | Targeted public health campaigns to increase awareness of AAS adverse effects such as hepatocellular carcinoma may be needed. | The cases indicate a need for AAS user communities and GPs to be aware of the possible effects and the risk for hepatocellular carcinoma. | Not Discussed | **⚫**  **⚫** |
| Van Hout, M.C.  2019  [**The use of sunless synthetic tanning products.**](https://www.taylorfrancis.com/chapters/edit/10.4324/9781315148328-11/use-sunless-synthetic-tanning-products-marie-claire-van-hout-jim-mcveigh)  Public Health Institute, Liverpool John Moores University, Liverpool, UK. | McVeigh, J. | **METHOD:** Review |  | - Increased socio-cultural values in the Western world are centred on bronzed, tanned skin equating with wealth, bodywork investment, attractiveness, and health and youthfulness. - Both genders are positively influenced by socio-cultural influences about tanning. - The use of untested and unregulated products available online | Monitoring and surveillance should continue to track product marketing and analyse contents as well as drug testing of seized products.  Synthetic tanning products should be included in clinical evaluation protocols and public health information (especially where the SunSmart message is prevalent). | It is critical that the risk of blood-borne virus transmission is effectively communicated to Melanotan injectors, in relation to both injecting and sexual behaviour. | Long-term clinical effects and user interactions should be investigated, as well as decision-making processes related to the introduction of use, continued use, and factors affecting the discontinuation of use.  A greater understanding of individual and peer group practices and beliefs of users of tanning products is recommended.  To establish and execute effective demand reduction interventions, it is essential to identify drivers of use and relevant practices. | **⚫** |
| Van de Ven, K.  2019  [**The modes of administration of anabolic-androgenic steroid users (AAS): are non-injecting people who use steroids overlooked?**](https://doi.org/10.1080/09687637.2019.1608910)  Social Policy Research Centre, University of New South Wales, Sydney. | Zahnow, R.  McVeigh, J.  Winstock, A. | **AIM: To** explore patterns of AAS use and to provides unique insights into the routes of administration of AAS users.  **METHOD:** Retrospective data analysis the Global Drug Survey 2015. | 1008 men who reported using AAS and at least one other psychoactive drug during their lifetime | - The majority of these AAS users   reported using only a single mode of administration (oral 35.84%; injection 35.62%).   - Non-injecting users who use psychoactive drugs not generally associated with injecting are unlikely to engage with NSPs which limits their access to harm reduction and medical advice. | An emphasis on assessing, tailored public health initiatives that include the AAS-using group in the implementation of them is recommended. | To reach non-injecting users, as well as to address the broader spectrum of medical services important to people who use AAS, a variety of interventions and health services are needed. | Future research is needed to explore why people engage in oral-only use and to explore barriers to accessing healthcare services for these users.  Given that NSPs are the most common way of delivering services and advice to people who use AAS, there is a need to better understand the proclivity for oral use only and find ways to meet this subset of users. | **⚫**  **⚫** |
| UKAD (UK Anti-doping)  2019  [**UKAD's Status Report on IPEDs in the UK.**](https://www.ukad.org.uk/sites/default/files/2020-01/UKAD%20IPED%20Report%20FINAL.pdf)  Fleetbank House, 2-6 Salisbury Square, London, EC4Y 8AE |  | **AIM:** To provide a  snapshot of the nature of IPEDs that are being used, where they come from and who takes them.  **METHOD:** Review |  | - To address this growing issue, it is important that a multi-agency, strategic approach is adopted to tackle the multiple influences for IPED use. - IPEDs must be considered a problem for anti-doping authorities and not just in sport. - The research highlights the growing public health issue of IPEDs based on copmlex influences such as media outlets (social media in particular), geographical, socioeconomic or criminality. | Collaboration between governments, law enforcement,  public health bodies,  anti-doping agencies,  educational institutions  and sports organisations  to address the issue of IPED use. | Proactive, inclusive, and constructive education is an important tool for encouraging clean sport, and it should be extended to minimise the use of IPEDs in general. | Future research should consider criminality, in terms of the scale, methods and implications both on society and in sport.  An investigation into young people’s motivations for use of IPEDs specifically whether the primary driver is sport or image is warranted. | **⚫**  **⚫** |
| Salinas, M.  2019  [**Polydrug use and polydrug markets amongst image and performance enhancing drug users: Implications for harm reduction interventions and drug policy.**](https://doi.org/10.1016/j.drugpo.2019.01.019)  Department of Sociology, Manchester Metropolitan University, Manchester, M15 6LL, UK. | Floodgate, W.  Ralphs, R. | **AIM:** To provide an in-depth account of the use and supply of IPEDs  **METHOD:** Qualitative: An ethnographic study of an independent, non-corporate ‘bodybuilding gym’, supplemented by 20 in-depth semi-structured interviews. | Gym member bodybuilders (*n=*17 males), personal trainer, gym owner and gym manager. | - Two main themes emerged: 1. Intersection of substance use: polydrug-using repertoires; and   2. Intersection of drug markets: the gym as a trading bazaar.   - Individuals who used IPEDs have concomitantly used diverted medicines, and more than half used illicit psychoactive drugs. - IPED use and supply were found to be linked to diverted medications and ‘traditional' recreational drugs. - IPED use was actively supported and facilitated by the gym and its staff members. | Punitive drug policy reform that seeks to minimise IPED markets must consider the possibility of social supply being displaced in favour of more commercially oriented dealing.  Criminalisation and stigmatisation of IPED users can result from harsher drug laws.  It is necessary to understand the possible negative consequences of reclassifying IPEDs. | The extensive poly-substance use identified by interviewees in this study necessitates a re-evaluation of existing IPED harm reduction guidance that considers the full spectrum of substances currently being used. | The emergence of more commercially minded suppliers and the dominance of social supply markets necessitates more in-depth research. | **⚫**  **⚫** |
| Richardson, A.  2019  [**Superheroes – Image and performance enhancing drug (IPED) use within the UK, social media and gym culture.**](https://doi.org/10.1016/j.jflm.2019.03.009)  Teesside University, UK. | Dixon, K.  Kean, J. | **AIM:** To explore the perspectives of steroid users regarding the social impact of steroid use on self and society.  **METHOD:** Qualitative interviews. | 24 IPED users | - Four main themes emerged: (1) Culture of Steroid Abuse, (2) Supply and Demand,   (3) Damage to Social Life,  (4) Next Generation of Users. | Not Discussed | Understanding the user's perspective and experiences are critical in assisting forensic and legal medicine practitioners. | Not Discussed | **⚫**  **⚫** |
| Richardson, A.  2019  [**Anabolic-androgenic steroids (AAS) users on AAS use: Negative effects, ‘code of silence’, and implications for forensic and medical professional.**](https://doi.org/10.1016/j.jflm.2019.101871)  Teesside University, UK. | Antonopoulos, G. A. | **AIM:** To offer an account of AAS users' perception of the negative aspects of AAS use.  **METHOD:** Qualitative interviews & Ethnography | Gym members, fighters, bodybuilder, AAS users. | - The substance reaction and range of negative experiences varies. - Medical advice is very rarely, if ever sought. - AAS users are a hard-to-reach population as they are unwilling to disclose their use and negative effects to others. | Not Discussed | Practitioners need to appreciate the diverse motivations and harms associated with steroid use, so that prevention and harm reduction can be informed. | Future research is needed to understand the dynamics of culture and how this effects AAS use.  Research to understand of the diverse motivations and harms associated with steroid use is warranted. | **⚫**  **⚫** |
| Mulrooney, K.  2019  [**Steroid Madness- Has the dark side of anabolic-androgenic steroids (AAS) been overstated?**](https://doi.org/10.1016/j.peh.2019.03.001)  University of New England (Australia). | van de Ven, K.  McVeigh, J. Centre for Public Health, Liverpool, United Kingdom. | **METHOD:** Review |  | - The overstating of negative effects of steroids has resulted in a distorted narrative, which may increase harms. - A more evidence-based approach to AAS use, free of morality and sensationalism, and based on clinical studies can mitigate harms. - The majority of adult AAS users do not suffer psychological dysfunction, hurt others, or die because of their use. | Policymakers and the public should not focus on isolated cases of harm or tragedy as this leads to misinformation. | Consideration for the reported positive effects whilst also reducing harms is crucial for practitioners. | Not Discussed | **⚫** |
| Mossman, J.A.  2019  [**The fertility fitness paradox of anabolic-androgenic steroid abuse in men.**](https://doi.org/10.1111/joim.12884)  Department of Ecology and Evolutionary Biology, Brown University, Providence, RI, USA. | Pacey, A.A.  Academic Unit of Reproductive & Developmental Medicine, Department of Oncology and Metabolism, University of Sheffield, Sheffield, UK | **METHOD:** Letter to the Editor |  | - As a result of AAS abuse, men risk reducing their probability becoming infertile. - The use of AAS and resulting negative effects create a direct conflict between their muscularity and masculinity. | Not Discussed | Educational outreach should  highlight the negative reproductive consequences of AAS use.  Efforts to inform doctors and AAS users about the clear connection between their 'perceived' fitness and their 'real' health is recommended to resolve this growing public health issue. | Not Discussed | **⚫**  **⚫** |
| McVeigh, J.  2019  [**Engaging with people who use image and performance enhancing drugs: One size does not fit all.**](https://doi.org/10.1016/j.drugpo.2019.05.016)  Centre for Public Health, Henry Cotton Campus, 15-21 Webster Street, Liverpool, United Kingdom. | N/A | **METHOD:** Response to commentary ‘The unintended consequences of emphasising blood-borne virus (BBV) in research on, and services for, people who inject image and performance enhancing drugs’ (Underwood, 2019 |  | Underwood (2019) voices the frustration of AAS and other IPED users who consider BBV as a distraction from their genuine concerns and not a significant issue.  Underwood (2019) relays that these individuals believe themselves to be experts of their own body and behaviours and that some published research is not an accurate representation of their lives.  The current commentary response reasserts the findings of research specific to this population.  In the UK evidence of BBVs among AAS users was reported in 2013.  The findings of this research are robust (Hope et al. 2013) and have been further supported by subsequent work. with additional evidence of undiagnosed hepatitis C as a significant concern. (Hope et al. 2016). | Not Discussed | Not Discussed | Researchers should ensure that they do not inadvertently create a barrier to service engagement for subgroups of users through the delivery of research findings. | **⚫**  **⚫**  **⚫** |
| Kotze, J.  2019  [**Boosting bodily capital: Maintaining masculinity, aesthetic pleasure and instrumental utility through the consumption of steroids.**](https://doi.org/10.1177%2F1469540519846196)  Teesside University, UK | Antonopoulos, A | **AIM:** To explore the contemporary importance attached to both attaining and maintaining both elements of a ‘boosted’ bodily capital**.**  **METHOD:** Qual Ethnography and Interviews | Male AAS users in a gym, bodybuilding competitions, fighting events and product promo events. | - The authors view the role of AAS in facilitating bodily capital as a means of hyper-conforming to neoliberalism’s cultural norms and values. - Steroids both facilitate the acquisition and maintenance of both dimensions of contemporary bodily capital. | Not Discussed | Not Discussed | Not Discussed | **⚫** |
| Hope, V.  2019  [**Infections and risk among people who use image and performance enhancing drugs.**](https://www.taylorfrancis.com/chapters/edit/10.4324/9781315148328-7/infections-risk-among-people-use-image-performance-enhancing-drugs-vivian-hope-jenny-iversen)  Public Health Institute, Liverpool John Moores University, Liverpool, UK. | Iversen, J. | **AIM: To** review what is known about the prevalence and range of both viral and bacterial infections among those who inject and use IPEDs; to examine what is known about the range and extent of injection and sexual risk behaviours about these infections.  **METHOD:** Review |  | - The injection of IPED drugs is associated with several public health concerns such as the risk of infections, including blood borne viral infections (HIV, hepatitis B and hepatitis C). - Early diagnosis and treatment will reduce the likelihood of onward transmission of BBVs occurring. - The data indicates that people using IPEDs may have an elevated risk of acquiring infections sexually when compared to the overall general population | The provision of and access to NSPs, sexual health services, condoms for IPED users. | Clinical and healthcare provisions such as blood testing services are required for the reduction of infections and to encourage diagnostic testing and treatment uptake for infections among injecting IPED users.  Care and treatment pathways for those who test positive is crucial.  Healthcare professionals need to be educated on IPED use and in particular, understand the stigma injecting IPED users may experience. | Further research is needed to design and develop appropriate responses for this group.  The potential role of pre and post exposure prophylaxis for HIV in preventing infection among gay and bisexual men who use IPEDs needs exploration. | **⚫**  **⚫** |
| Hill, S.A.  2019  [**Pharmacological effects and safety monitoring of anabolic androgenic steroid use: differing perceptions between users and healthcare professionals.**](https://doi.org/10.1177/2042098619855291)  York Hospitals NHS  Foundation Trust, York,  North Yorkshire, UK. | Waring, S.W. | **AIM: T**o ascertain how AAS abusers interact  with healthcare providers, and self-reported knowledge among doctors regarding the healthcare effects and need for monitoring of AAS users.  **METHOD:** Two Surveys: 1) bodybuilders/AAS users; 2) Doctors. | 216 AAS users.  134 Doctors. | - Barriers to healthcare experienced by AAS users included: fear of being judged, perceived lack of AAS knowledge among GPs, and felt AAS use on their health records was detrimental. - Most GPs would not provide blood tests for people who used IPEDs and did not feel confident interpreting results. | Not Discussed | Barriers to healthcare experienced by AAS users’ needs to be addressed by HCPs to minimize adverse health outcomes of use. | Future research is recommended to examine if these findings are generalizable to doctors working in other regions. | **⚫** |
| Henning, A.  2019  [**"Yay, Another Lady Starting a Log!": Women's Fitness Doping and the Gendered Space of an Online Doping Forum.**](https://doi.org/10.1177%2F2167479519896326)  Faculty of Health Sciences and Sport, University of Stirling, Scotland. | Andreasson, J. | **AIM:** To investigate and dissect the meanings attached to female IPED use and how fitness doping can be understood in terms of gender and spatiality, and what implications this has for women’s communicative engagement with one another within the online forum.  **METHOD:** Qualitative case study approach and netnographic methodology. | Female AAS users | - Three themes emerged: Motivations for Engaging in the Forum; Experimenting; and renegotiating a Gendered Space and Practice. - Even though the forum offers a platform for women to express their own specific experiences, the degree to which the discussions reflect women's experiences is limited. - Male experiences and voices often dominated the discussions which can impede the development of women’s community of practice (CofP) - Women seeking guidance or other women's perspectives must navigate through men's contributions. | Not Discussed | Not Discussed | Future research is required to explore the impact these changes have on women’s fitness doping practices and a changing doping demography in terms of prevention and HR. | **⚫** |
| Harvey, O.  2019  [**Social Work Implications of Anabolic–Androgenic Steroid Use, Particularly Among Young People: A Literature Review.**](https://doi.org/10.1080/09503153.2019.1653450)  Faculty of Health and Social Sciences, Bournemouth University. | Parrish, M. | **AIM: To** provide an overview of the physical and psychological harms associated  with AAS use; to identify the motivations for recreational AAS use; to identify the risks of AAS use to young people; and to explore the implications for social workers working with those at risk of  AAS use.  **METHOD:** Literature Review |  | - Social workers should advise AAS users and families of behavioural changes following AAS use that may result in aggression. - Social workers awareness of AAS patterns of use is significant for the identification of potential vulnerabilities and to identify appropriate supports available. - Knowledge of the legal status of AAS in their region/country is important for social workers. - Risk assessments by social workers must be balanced and not focus on the so-called ‘roid-rage’ view held by the public. | Not Discussed | Social workers are required to have sufficient expertise on the complexities of AAS use to establish their credibility. | Future research is required to explore the potential risks families and partners of AAS users may face.  A significant amount of research regarding psychosocial factors related to young people at risk of AAS use is required. | **⚫**  **⚫**  **⚫**  **⚫** |
| Harvey, O.  2019  [**Support for people who use Anabolic Androgenic Steroids: A Systematic Scoping Review into what they want and what they Access.**](https://doi.org/10.1186/s12889-019-7288-x)  Faculty of Health and Social Sciences, Bournemouth University. | Keen, S.  Parrish, M.  van Teijlingen, E. | **AIM:** To explore and identify the nature and scope of information and  support both accessed and wanted by non-prescribed AAS users.  **METHOD:** Scoping Review |  | - How to inject, effectiveness of substances, dosages, and side effects of AAS use were the type of information AAS users seek. - Support was sought from NSPs, medical professionals, friends, dealers, and online. - Peer advice was preferred over professional advice. | Not Discussed | Support should be tailored to the needs of AAS users.  Health care practitioners should be sensitive to AAS users’ perceptions their use and the related stigma of being associated with other IDUs to facilitate effective engagement.  It is pertinent that HCPs have in-depth knowledge of benefits, harms, and range of drugs available for PCT | Research is warranted to gain understanding of AAS users drug use, stigma experienced and support needs, specifically female and transgender AAS users. | **⚫** |
| Harris, M.A.  2019  [**Symptoms of Muscle Dysmorphia Between Users of Anabolic Androgenic Steroids with Varying Usage and Bodybuilding Experience**.](https://psycnet.apa.org/doi/10.1027/2512-8442/a000023)  Department of Applied Psychology, Cardiff Metropolitan University, UK. | Alwyn, T.  Dunn, M. | **AIM:** To focus on the extent to which MD may precipitate or perpetuate the use of AAS.  **METHOD:** Cross-sectional survey | Male bodybuilders (n= 57 users and 51 non-users), Wales, UK. | - MD, bodybuilding, and AAS use have a more complex relationship than previously reported. - MD may force vulnerable people to initiate a AAS use. - There were no variations in MD symptoms recorded between AAS users with different lengths of AAS exposure. - The study indicates that MD could influence both the initiation and maintenance of AAS use. | Not Discussed | Treatment interventions targeted at maladaptive body image concerns (i.e., MD symptomology) may be effective in reducing or preventing AAS use.  Individuals with body image issues may benefit from a counterintuitive intervention strategy that encourages them to engage in weight-resistance exercise in a supportive social setting rather than a weight-training focused gym. | Longitudinal research is recommended.  The relationship between MD and AAS use warrants exploration. | **⚫** |
| Hall, A.  2019  [**The (online) supply of illicit lifestyle medicines: a criminological study**.](https://www.taylorfrancis.com/chapters/edit/10.4324/9781315148328-13/online-supply-illicit-lifestyle-medicines-alexandra-hall-georgios-antonopoulos?context=ubx&refId=f7f90081-f17a-4cd1-9c8f-02bd90431be0)  Northumbria University, Newcastle. | Antonopoulos, G. A. | **AIM:** To provide an empirically grounded social scientific analysis of the nature and dynamics of the trade of enhancement drugs in the UK.  **METHOD:** qualitative methods both online and offline settings |  | - This chapter illustrates how the supply of illicit medicines is entrenched in global variations in IPRS, the blurred boundaries of the legitimate and illegitimate pharmaceutical industries, the global free market and transnational trade relations. - There is a clear inextricable link between online and offline trade. - The global ease of access to internet and communications has facilitated criminal actors’ expansion in trade and opportunities. - Modern ICTs, the internet, and e-commerce promotes the production and distribution of illicit medicines. | Not Discussed | Not Discussed | Not Discussed | **⚫** |
| Glass, R.  2019  [**Secondary distribution of injecting equipment obtained from needle and syringe programmes by people injecting image and performance enhancing drugs: England and Wales, 2012-15.**](https://doi.org/10.1016/j.drugalcdep.2018.11.021)  National Infection Service, Public Health England, London, UK. | Hope, V. D.  Njoroge, J.  Edmundson, C.  Smith, J.  McVeigh, J.  Parry, J.  Desai, M. | **AIM:** To estimate the extent of secondary distribution of injecting equipment amongst people who inject IPEDs.  **METHOD:** Retrospective data analysis | IPED injectors | - Secondary distribution of injecting equipment obtained from NSPs is common. - 154 people collected equipment for 639-1569 other IPED injectors. - More people received injecting equipment indirectly through secondary distribution. - Collecting injecting equipment for others was associated with sharing equipment and SSTI symptoms. - Significant need for NSPs to address secondary distribution appropriately. | Not Discussed | By including those collecting as peer educators and using social media and online networking for health promotion, secondary distribution could be beneficial.  NSPs could provide online ordering of injecting supplies, as well as injecting practices education.  Innovative outreach approaches among IPED user communities that offer unique access for delivery of both NSPs, and education is recommended. | Research is recommended to highlight the significance of improved approaches and alternative platforms for delivery of harm reduction interventions for people injecting IPEDs. | **⚫**  ⚫ |
| Germain, J.  2019  [**One size does not fit all: Tackling the issue of weight-loss drug use.**](https://www.taylorfrancis.com/chapters/edit/10.4324/9781315148328-22/one-size-fit-jennifer-germain-charlotte-mclean-conan-leavey)  Public Health Institute, Liverpool John Moores University, Liverpool, UK. | McLean, C.  Leavey, C. | **AIM:** To argue against a ‘typical’ weight-loss drug user.  **METHOD:** Review |  | - A ‘one size fits all’ model for weight-loss drug use harm reduction may be ineffective. | There is no ‘typical’ user, and a one size fits all harm reduction model is not effective. | Interventions should be identified that are not based on a user typology but focus on vulnerable users such as those with significant body image disruption or who are engaging in disordered eating. | Further research is needed to gain more understanding of the behaviours, motivations, and characteristics of this population. | **⚫** |
| Dreyer, B.A.  2019  [**Melanotan-induced priapism: a hard-earned tan.**](https://doi.org/10.1136/bcr-2018-227644)  Urology, Queen Elizabeth  University Hospital, Glasgow, UK. | Amer, T.  Fraser, M. | **METHOD:** Clinical case report. | 41-year-old male melanotan user, Scotland. | - Following subcutaneous injection of ‘melanotan’ into the lower abdomen, painful, unrelenting penile erection lasting 22 hours. - Attempted to stop the erection with ice application to penis. - Bilateral aspiration of blood from the corpa cavernosa carried out. - Patient did not know what dosage was in each phial. - Patient was unaware of side-effects, labelling did not provide this information. | Not Discussed | Future therapeutic application of tanning agents will need to consider this potential life altering complication. | - Not Discussed | **⚫**  **⚫** |
| Corazza, O.  2019  [**The Emergence of Exercise Addiction, Body Dysmorphic Disorder, and other Image-related psychopathological correlates in Fitness Settings: a cross sectional study.**](https://doi.org/10.1371/journal.pone.0213060)  Centre for Clinical & Health Research Services, School of Life and Medical Sciences, University of Hertfordshire, Hatfield, United Kingdom. | Simonato, P.  Demetrovics, Z.  Mooney, R.  van de Ven, K.  Roman-Urrestarazu, A.  Racmolnar, L.  De Luca, I.  Cinosi, E.  Santacroce, R.  Marini, M.  Wellsted, D.  Sullivan, K.  Bersani, G.  Martinotti, G. | **AIM:** To explore the diffusion of EA in fitness settings in the United Kingdom, Italy, Netherlands, Hungary, and the previously unexplored association with appearance anxiety, BDD, self-esteem and the use of fitness supplements.  **METHOD:** Cross-sectional survey | Fitness club members, aged 18+ | - Exercise addiction was present in fitness settings in the research. - EA was associated with appearance anxiety, low self-esteem, and the use of a variety of fitness supplements taken without medical consultation. - Fitness goals are influenced by a person’s perception of their reflection in the mirror, or the number of “likes” for pictures on social networking sites. - Consideration for aesthetic idealisation, rather than for wellbeing, or a hedonistic pursuit of pleasure is recommended. | Targeted prevention strategies and policy responses are urgently required to address this at risk-population.  as well as the need  to inform who  could identify potential patients requiring referral to specialized treatment. | Clinicians and professionals working in both sport and aesthetics urgently require these targeted prevention strategies and policy responses. | Future research investigating impulsivity, compulsivity, and perception of the self, both in general and clinical populations is recommended for the development of new knowledge and effective treatment. | **⚫** |
| Coomber, R.  2019  [**The supply of image and performance enhancing drugs (IPED) to local non-elite users in England: Resilient traditional and newly emergent methods.**](https://www.taylorfrancis.com/chapters/edit/10.4324/9781315148328-17/supply-image-performance-enhancing-drugs-iped-local-non-elite-users-england-ross-coomber-mike-salinas)  Department of Sociology, Social Policy and Criminology, University of Liverpool. | Salinas, M. | **AIM:** Review of two research studies on IPED supply.  **METHOD:** Review of 2 studies: Study A – ethnography & interviews; Study B –Qualitative interviews and substance (IPEDs) analysis | IPED users and suppliers | - IPED users access substances from fellow gym-goers/friends or gym staff and it is largely normalised within gym settings. - Many IPED users reluctant to buy online -considered unreliable, potentially harmful, and expensive. - Most products do not contain what the packaging indicates. | Not Discussed | Harm reduction workers need to be more proactive in outlining the poor quality and dangers of substances sourced by IPED users. | Not Discussed | **⚫** |
| Brennan, R.  2019  [**“Beauty through the eye of a needle” - An online study of the practices and beliefs of people who inject performance and image enhancing drugs (PIEDs).**](https://www.taylorfrancis.com/chapters/edit/10.4324/9781315148328-10/beauty-eye-needle-rebekah-brennan-john-wells-marie-claire-van-hout?context=ubx&refId=50c55447-bfb3-421c-a667-e3465b7d3deb)  University College Cork, Ireland.  **Co-authors: UK** | Wells, J.S.G.  Van Hout, M.C.  . | **AIM:** To explore and describe experiences, beliefs and values of individuals who inject PIEDs.  **METHOD:** Netnography | Injecting IPED users in online forum spaces | - People who inject IPEDs often educate themselves on how to inject sometimes injecting cocktails of substances subcutaneously. - Self-phlebotomy was reported as a solution to high blood pressure and excess red blood cells. - Needle fixation and   injecting pleasure was identified and fearless injecting a symbol of masculinity.   - Increased autonomy and selfhood through injecting was reported by females. | Targeted harm reduction interventions aimed at protecting the safety and health of injecting IPED users is recommended.  Consideration of the benefits of online interventions is warranted. | HCPs are advised to adopt an empathic and non-judgemental approach when engaging with individuals who inject IPEDs.  Guidance for HCPs on the presentations of harms associated bloodletting and self-injection of Botox and dermal fillers is necessary. | Not Discussed | **⚫**  **⚫**  **⚫**  **⚫**  **⚫** |
| Boardley, I.  2019  [**Licit forms of performance enhancement and possible links with IPED use: current knowledge and future directions.**](https://www.taylorfrancis.com/chapters/edit/10.4324/9781315148328-6/licit-forms-performance-enhancement-possible-links-iped-use-ian-boardley?context=ubx&refId=7ac81bdc-c2f3-409a-973a-5d7823156256)  School of Sport, Exercise & Rehabilitation Sciences, University of Birmingham, Birmingham, UK. |  | **AIM:** To critically explore the gateway hypothesis of doping in sport and exercise (GHDSE) and the incremental model of doping behaviour (IMDB) focusing on processes proposing why using licit forms of performance enhancement may increase susceptibility to subsequently using illicit forms.  **METHOD:** Review |  | - The review suggests that the positive link between licit and illicit forms of performance enhancement is meaningful and robust. - The reliance on cross-sectional designs to date denotes that no claims regarding temporal sequencing and causality can currently be made. | Firmer recommendations for possible interventions and policies to limit PEDs in sport are needed. | Practitioners could present licit forms of performance enhancement as alternatives to doping.  A ‘food first’ approach to divert athletes away from PEDs. | Longitudinal research to track athletes use of licit and illicit means of performance enhancement over time, and analyses of the temporal sequencing present is required. | **⚫**  **⚫** |
| Bates, G.  2019  [**Preventing image and performance enhancing drug use: It’s not all chalk and talk.**](https://www.taylorfrancis.com/chapters/edit/10.4324/9781315148328-20/preventing-image-performance-enhancing-drug-use-geoff-bates-susan-backhouse?context=ubx&refId=02d97f7e-09e1-4bde-84ca-8c99a85f1778)  Public Health Institute, Liverpool John Moores University, Liverpool, England. | Backhouse, S. | **METHOD:** Review |  | - A ‘one size fits all’ approach will not be successful in either sport or public health environments. - There is a need for multi-layered interventions that recognise the variation and complexity characteristic of IPED use and the lives of those who use them. - In prisons or settings where harm reduction and treatment approaches are not supported is a concern as their risk of harm without intervention is exacerbated. | Delivery of an integrated range of interventions and policies based on scientific evidence to prevent initiation to and prevalence of IPED use are needed. | The complex factors that make children, youth, and adults vulnerable to IPED use and other risky behaviours require specific interventions for prevention. | Future research is needed to inform policy and interventions to prevent initiation of IPED uses. | **⚫**  **⚫** |
| Bates, G.  2019  [**Treatments for people who use anabolic androgenic steroids: a scoping review.**](https://doi.org/10.1186/s12954-019-0343-1)  Public Health Institute, Liverpool John Moores University, Liverpool, England. | Van Hout, M. C.  Teck, J. T. W.  McVeigh, J. | **AIM:** To identify and describe what is known about interventions that aim to support and achieve cessation of AAS and treat and prevent associated health problems.  **METHOD:** Scoping review | AAS users | - Evidence on interventions to support cessation of AAS use or responding to dependence is scant. - Evidence of engagement with AAS users, delivery of psychosocial interventions, and harm reduction interventions alongside, or following, treatment is limited. - Evidence of treatment for dependence, managing withdrawal, or initiating behaviour change in users is scarce. | Not Discussed | Not Discussed | Consistent reporting of engagement assessment, intervention and outcome of treatment is required. | **⚫** |
| Bates, G.  2019  [**A systematic review investigating the behaviour change strategies in interventions to prevent misuse of anabolic steroids.**](https://doi.org/10.1177/1359105317737607)  Public Health Institute, Liverpool John Moores University, Liverpool, England. | Begley, E.  Tod, D.  Jones, L.  Leavey, C.  McVeigh, J. | **AIM: To** examine intervention effectiveness of strategies to prevent IPED use.  **METHOD:** Systematic review. |  | - Using the EPHPP tool three studies were rated strong, five studies were rated moderate, and nine studies were rated weak. - Evidence on how to reduce use of AAS is scarce.   There is a need to respond to the different issues of the use of AAS outside of sporting environments and in adult populations.   - Strategies to tackle AAS use will be informed by increased understanding of factors that influence decision-making, and the transferability of evidence from other relevant fields. | - Not Discussed | Suitable interventions are required that support IPED use within the general population. | To establish the evidence base in this area, a more consistent  and rigorous approach to the development and reporting of interventions is required. | **⚫**  **⚫** |
| Andreasson, J. e al.  2019  [**Glocal fitness doping: Policy, practice and prevention in the United States and Sweden.**](https://doi.org/10.1016/j.peh.2018.11.001)  Linnaeus University, Department of Sport Science, 39182 Kalmar, Sweden.  **Co-author: UK** | Henning, A.  . | **AIM:** To investigate and compare how fitness doping can be understood in relation to, and how it is affected by, different national and local contexts.  **METHOD:** Comparative analysis of empirical data from Sweden and United States. |  | - Two themes emerged: Individual freedom and doping in the U.S; and Fitness doping as a societal problem in Sweden. - Sweden’s choices reflect the priority for protecting the collective good over individual pursuits. - The U.S. does not police outside formally governed competitions in sports or in criminal contexts. - U.S. bodybuilders do not feel targeted for their appearance in the same ways, illustrating the priority of individual choice | The gap between the global and the local level requires attention by researchers and policymakers. | Glocal fitness doping needs to be understood as a process through which global ideals, organisations, and more contribute to influencing local and national prevention policies and cultures, and vice versa. | The gap between the global and the local level requires attention by researchers and policymakers. | **⚫**  **⚫** |
| Zahnow, R.  2018  [**Identifying a typology of men who use anabolic androgenic steroids (AAS).**](https://doi.org/10.1016/j.drugpo.2018.02.022)  School of Social Science, The University of Queensland, St Lucia, Brisbane, Queensland, 4072, Australia. | McVeigh, J.  Bates, G.  Hope, V. D.  Kean, J.  Campbell, J.  Smith, J. | **AIM:** To identify typologies of people who use AAS and examine variations in motivations for AAS use across types  **METHOD:** Cluster Analysis; Survey at NSPs and Online Fora | 611 men who use AAS | - Study developed a quantitative typology of AAS users. - Four types: YOLO, Wellbeing, Expert and Athlete types. - YOLO type is young, inexperienced, with high levels of risk; Wellbeing frequent the gym, concerns with image and wellbeing, less risky; Athlete type were more likely to be older, use of AAS and other IPEDs; Expert type minimal AAS and IPED use, low alcohol consumption, low risk. | Not Discussed | Harm minimisation services are required to be equipped to  disseminate information about safe intra-muscular injecting and ensuring needle disposal sites are accessible. | Further analyses of routine data and survey data together with diverse datasets is recommended. | **⚫** |
| Teck, J.  2018  [**Tracking internet interest in anabolic-androgenic steroids using Google Trends.**](https://doi.org/10.1016/j.drugpo.2017.11.001)  MRC/CSO Social and Public Health Sciences Unit, University of Glasgow, United Kingdom. | McCann, M. | **AIM:** To assess the feasibility of using Google Trends to supplement what  we know about AAS related behaviour.  **METHOD:** Google Trends Time Series Analysis |  | - A clear and consistent seasonal variation with 9 of the 10 compounds reaching statistical significance was present. - From April to July (bulimic seasonality) peaks of search interest were noted. - The study may support the idea that AAS use is part of health and beauty consumerism rather than primarily related to body dysmorphia or a substance use disorder. | Harm reduction policies need to address the underlying neoliberal and consumerism driven risk normalisation underlying AAS use. | Health promotion, prevention, and harm reduction services may be more effective if targeted during periods of increased internet search interest. | Google Trend data should be interpreted with caution, and potential work involving triangulation with other data sources may make this approach more useful. | **⚫**  ⚫ |
| Scottish Drug Forum Working Group on IPEDs  2018  **Imaging and Performance Enhancing Drugs (IPEDS) in Scotland.**  91 Mitchell St, Glasgow G1 3LN |  | **AIM:** To capture our current understanding of IPED use, people’s motivations for use, associated harms and services responses in Scotland to date.  **METHOD:** Review |  | ***Four Issues Identified:***  1. A lack of data and research  2. Need for specialist IPED Provision  3. Need for Links to health services and endocrinology.  4. The role of gyms – need to be viewed as good practice and not as supply route | Responses should evolve to influence the Scottish Government's drug policy and responses under the Sexual Health and Blood Borne Viruses frameworks. | Health boards in each different area should consider the demand for a specialist IPED service provision.  Endocrinologists require training provision to ensure that there is an appropriate level of knowledge of IPED use and associated problems. A formal link between specialist IPED services and endocrinology needs to be established. | Research is needed to improve routine data collection around IPED use to provide regular data on usage patterns and trends.  A prevalence study is warranted, to ascertain an accurate picture of current IPED use. | **⚫**  **⚫**  **⚫**  **⚫**  **⚫**  ⚫ |
| Harvey, O.  2018  [**‘Shades of Grey’: The Ethics of Social Work Practice in Relation to Un-prescribed Anabolic Androgenic Steroid Use.**](https://doi.org/10.1080/09503153.2018.1510480)  Faculty of Health and Social Sciences, Bournemouth University, Bournemouth, UK. | N/A | **AIM:** To reflect on some of the ethical dilemmas that social workers face when assessing risk in relation to those using substances  **METHOD:** Review |  | - Most people who use AAS are not vulnerable because of their use. - Health risks may increase if one become dependent on AAS. - No evidence that individuals are unable to manage daily living whilst using AAS. - Social workers should be aware some groups of people who are more at risk to starting AAS use. | Not Discussed | Social work practitioners should be able to understand the motivations for AAS use so that they discuss the associated risks. | Further qualitative research to understand the reasons for initiation to use and support to help people stop using AAS is warranted. | **⚫**  **⚫**  **⚫**  ⚫ |
| Greenway, C.W.  2018  [**A qualitative study of the motivations for anabolic-androgenic steroid use: The role of muscle dysmorphia and self-esteem in long-term users.**](https://doi.org/10.1016/j.peh.2018.02.002)  School of Psychology, University of Wales Trinity Saint David, College Road, Carmarthen, SA31 3EP, United Kingdom | Price, C. | **AIM: T**o determine whether MD symptoms and low self-esteem feature in the descriptions of long-term AAS users’ motivations for initiation and continued use.  **METHOD:** Qualitative Interviews | Male Weightlifters | Motivations for Use reported were self-esteem, body dissatisfaction and MD symptoms.   - MD Symptoms developed in some because of them feeling ‘physically inferior’ to others. - The primary motivator for use among those who began using AAS in their teens was social acceptance (with low self-esteem). - Frustration with training (and a drive for muscularity) was the motivation for those who started using in their late 20s, - Inability to cease AAS use was due to fear of losing gains; psychological addiction to the way AAS made them feel; and age-related benefits to stave off ageing. | Future initiatives will need to focus their attention on educational programmes that build self-esteem and improve social skills in schools, universities, and community projects. | Relevant age groups should be targeted by health practitioners with effective interventions and treatments.  Future initiatives may wish to assist those who depend on the positive effects of AAS to explore alternative sources of social reward and esteem.  Future programmes will benefit from going into gyms and NEPs to provide training and awareness on the misconceptions surrounding the psychological benefits of AAS. | Future research exploring the age at which men start using AAS may highlight age-specific motivations.  An examination of the development of MD post AAS use in current and former users is warranted. | ⚫  **⚫** |
| Chester, N.  2018  [**Drug Use in Society and the Impact on the Anti-Doping Movement.**](https://www.taylorfrancis.com/chapters/edit/10.4324/9781315222790-26/drug-use-society-impact-anti-doping-movement-neil-chester-jim-mcveigh)  Faculty of Science, Sport and Exercise Sciences, Liverpool John Moores University. | McVeigh, J. | **AIM:** To examine the use of IPEDs in the general population  **METHOD:** Review |  | - The issue of doping and drug use highlights complex societal issues which cannot be examined in isolation. - Athletes and the wider society may use drugs for a wide variety of non-therapeutic reasons, such as for recreation and/or enhancement purposes. - The continually shifting societal values towards youthfulness and immediate gratification further enhances the demand for and acceptance of enhancement technologies | It is essential that the anti-doping movement continues to debate the issues relating to  PIED use within the context of societal drug use. | Not Discussed | Not Discussed | **⚫**  **⚫** |
| Brennan, R.  2018  [**"Bloodletting"-Self-phlebotomy in injecting anabolic-androgenic steroids within performance and image enhancing drug (PIED) culture.**](https://doi.org/10.1016/j.drugpo.2018.02.011)  School of Health Science, Waterford Institute of Technology, Cork Road Waterford, Ireland.  **Co-authors: UK** | Wells, J. S. G.  Van Hout, M.C.  . | **AIM: T**o theoretically explore how a practice such as self-phlebotomy may become normalised in the online discussion forum space.  **METHOD:** Netnography | Online Forum Members | - Three themes emerged: motivation to self-bleed; Bloodletting practices; and Health harms. - Motivation to ‘self-bleed’ were grounded in a high red blood cell count or (perceived) symptoms of high blood pressure. - No harms were reported by forum discussants or awareness of potential complications. |  |  | More research is needed in this field, and it would help healthcare workers, treatment providers, and policymakers, especially in terms of evidence-based and focused harm reduction policies and successful public health initiatives. | **⚫**  ⚫ |
| Brennan, R.  2018  [**“Raw juicing” – an online study of the home manufacture of anabolic androgenic steroids (AAS) for injection in contemporary performance and image enhancement (PIED) culture.**](https://doi.org/10.1016/j.peh.2017.11.001)  School of Health Science, Waterford Institute of Technology, Cork Road Waterford, Ireland.  **Co-authors: UK** | Wells, J. S. G.  Van Hout, M.C.  . | **AIM:** To explore the home manufacture of anabolic androgenic steroids (AAS) for injection, known as ‘homebrewing  **METHOD:** Netnography | Online Forum Members | - Homebrewing motivations were grounded in the circumventing of unreliable online sourcing of AAS products, financial losses and potential harms associated with contaminated and counterfeit injectables. - The potential for sterility and dosing issues, injecting harms, and isolation from health services were identified areas of concern. |  |  | Health-care professionals, treatment providers, and policymakers will benefit from further research in this field, especially as it relates to evidence-based and focused harm reduction strategies and successful public health initiatives. | **⚫** |
| Boardley, I.D.  2018  [**Development of moral disengagement and self-regulatory efficacy assessments relevant to doping in sport and exercise.**](https://doi.org/10.1016/j.psychsport.2018.01.007)  University of Birmingham, UK. | Smith, A. L.  Mills, J.  Grix, J.  Wynne, C.  Wilkins, L. | **AIM:** To develop Moral Disengagement (MD) and Self-Regulatory Efficacy (SRE) instruments relevant to  doping in sport and exercise and provide evidence for the validity and reliability of instrument scores.  **METHOD:** Cross-sectional, correlational |  | - Three psychometric instruments relevant to the psychology of doping were developed. - Factor analyses identified the final items and dimensional structures for the Doping Moral Disengagement Scale (DMDS), Doping Moral Disengagement Scale–Short (DMDS–S) and Doping Self-Regulatory Efficacy Scale (DSRES). - The DMDS has 6 lower- and 1 higher-order factors. - The DMDS-S and DSRES are unidimensional. - These structures were invariant by sport/exercise context and sex. - Evidence was also provided to support the scales’ external validity, test-retest reliability, and score stability. - The scales are the first doping-contextualized instruments developed for use with both sport and exercise populations. | Not Discussed | Not Discussed | Further research is recommended to further evaluate the instruments employed. | **⚫**  **⚫** |
| Bates, G.  2018  [**An evidence-based socioecological framework to understand men’s use of anabolic androgenic steroids and inform interventions in this area.**](https://doi.org/10.1080/09687637.2018.1488947)  Public Health Institute, Liverpool John Moores University, Liverpool, England. | Tod, D.  Leavey, C.  McVeigh, J. | **AIM:** To discuss the different influences on AAS use and how these influences interact to effect decision-making in one framework.  **METHOD: Review** | AAS users | - Socioecological framework suggests that the interaction of many factors at the individual, social network, institutional, community, and   societal levels that are likely to change over time influences the use of AAS.   - By incorporating the socioecological and typology frameworks to identify factors that are influential for different groups will result in an increased understanding of AAS use. | An increased understanding of AAS use can inform the responses of health professionals and policy makers. | Not Discussed | Further research to develop our understanding of the influences of peers,  social networks, and norms about AAS, muscularity and masculinity,  particularly where  AAS are associated with success or social rewards is warranted. | **⚫**  ⚫ |
| Angell, P.G.  2018  [**Acute cardiovascular responses to resistance exercise in anabolic steroids users: A preliminary investigation.**](https://doi.org/10.1016/j.scispo.2018.05.003)  Liverpool Hope University, L16 9JD Liverpool, UK. | Green, D. J.  Lord, R.  Gaze, D.  Whyte, G.  George, K. P. | **AIM:** To assess cardiac function and biomarker responses to a high intensity whole-body resistance exercise session in age matched AS users and non-AS users (NAS).  **METHOD:** Resistance exercise session with echocardiographic measures and cardiac  biomarkers taken pre and post exercise | Males, strength trainers, from gyms and NSPs | - A reduction in several indices of diastolic function (E’, E’: A’ and E: A) post-exercise was not mediated by AS use. - Wholesale changes in biomarkers of cardiac damage were not produced by an acute bout of resistance exercise. - There was no significant effect of exerciser AS use on E: E’. - Changes to diastolic driving forces and active relaxation can occur independently of each other | Not Discussed | An increase in the number of post-exercise testing will aid in better understanding of the potential appearance of cardiac biomarkers after resistance exercise. | Future research focusing on incorporating left atrial measure to further help the understanding of changes to diastolic function is warranted. | **⚫** |
| Ainsworth, N.P.  2018  [**Being in control? A thematic content analysis of 14 in-depth interviews with 2,4-dinitrophenol user.**](https://doi.org/10.1016/j.drugpo.2017.12.012)  Kingston University, Penrhyn Road, Kingston-Upon-Thames, Surrey, KT1 2EE, UK. | Vargo, E. J.  Petroczi, A. | **AIM:** To explore 2,4-DNP users’ attitudes towards the Internet as a tool, perceived attitudes of in-groups and out-groups towards their 2,4-DNP use, as well as exploring 2,4-DNP use through participants’ attitudes towards the compound.  **METHOD:** Qualitative interviews | Novel & experienced DNP users on bodybuilding forums. | - The element of control over every aspect of the users’ life appears to be a key component for the successful risk-management of 2,4-DNP use. - The study highlighted the importance of creating and maintaining community relations with the target population. | Policies and interventions can be better informed by understanding users’ motives and decision-making processes. Policy creation could possibly occur through well-conducted participant action research initiatives | Health care practices are advised to adopt a non-judgemental approach and avoid additional marginalisation of 2,4-DNP users. | Cross-country research to determine if cultural background influences an individuals’ approach PED use in particular, fat burners such as 2,4-DNP) is recommended.  Future research in gyms and other online platforms of 2,4-DNP use is warranted. | **⚫** |
| Zahnow, R.  2017  [**Adverse Effects, Health Service Engagement, and Service Satisfaction Among Anabolic Androgenic Steroid Users.**](https://doi.org/10.1177%2F0091450917694268)  Institute for Social Science Research, The University of Queensland, St. Lucia, Queensland, Australia.  **Co-authors: UK** | McVeigh, J.  Ferris, J.  Winstock, A. | **AIM:** To explore factors associated with health service engagement and treatments related to service satisfaction among AAS users.  **METHOD:** Retrospective data analysis of the GDS | AAS Users | - 35% reported seeking advice from GP due to AAS related adverse effects with the majority reluctant to do so. - Sexual function issues were a driver for men to seek GP treatment. - Those AAS user that did seek GP and medical advice were in the older age group. - Diabetes tests and mood assessments were associated with higher ratings of service helpfulness and one treatment. - Hepatitis B/C and HIV screening were associated with higher ratings of overall experience with the doctor. | The incidence and severity of these AAS-related harms may be ameliorated if users engage with health services when concerns first arise. | Providers of health care must show that they are both non-judgmental and informed about the use of AAS.  The benefits of AAS must be acknowledged by health service providers. | Not Discussed | **⚫**  ⚫ |
| Mooney, R.  2017  [**The use of supplements and performance and image enhancing drugs in fitness settings: A exploratory cross‐sectional investigation in the United Kingdom.**](https://doi.org/10.1002/hup.2619)  Psychology Department, University of Hertfordshire, Hatfield, UK. | Simonato, P.  Ruparelia, R.  Roman-Urrestarazu, A.  Martinotti, G.  Corazza, O | **AIM:** To explore the use of products to enhance performance alongside exposure to exercise addiction, appearance anxiety and self‐esteem in fitness settings  **METHOD:** Cross-sectional survey |  | - Males were the majority of those who reported product use to achieve their fitness goals. - Females were more inclined to use weight loss products. - Experience side effects was not stated in relation to the consumption of specific substances. - Severe implications regarding polypharmacy may occur in the presence of physical illness which might need medication, | Healthcare professionals and regulatory authorities are urgently required to safeguard public health.  Implementation of restrictions for false or misleading advertising is warranted. | Not Discussed | Further investigation of the variety of side effects reported is needed.  More targeted studies are needed with larger sample sizes focusing on appearance anxiety and low body satisfaction. | **⚫**  **⚫**  **⚫** |
| Miller, R.  2017  **Building Bodies: Investigating Image and Performance Enhancing Drugs (IPEDs) Use in Scotland.**  Senior Research Officer, The Scottish Government | N/A | **AIM:** To investigate Image and Performance Enhancing Drugs (IPEDs) Use in Scotland  **METHOD:** Review |  | - The implications of the rise in IPED use in the UK and Scotland, particularly in relation to the prevalence of BBVs amongst IPED users is a public health concern. - Dedicated IPED services have potential to promote engagement and reduce harms | The development of existing and planned IPED services in Scotland requires continued support.  The development of a formal Scottish strategy relating to IPED services is warranted.  Ongoing evaluations of dedicated IPED services available in Scotland should be considered by policymakers. | Relationships between local services (e.g., mental health, sexual health services, drug, and alcohol services) and IPED services could be strengthened.  Additional training for GPs on IPEDs is recommended to encourage a non-judgmental and well-informed service for IPED users and to bridge the gap that exists between IPED users and GPs.  Peer-led IPED information and harm reduction networks are encouraged. | - A Scotland-wide study to ascertain prevalence, distribution and drug use patterns including use in prison. - Information on the market including drug composition.   and the role of the internet   - Harms including mental health issues and BBV, body dysmorphia, and long-term herms | **⚫**  **⚫**  ⚫ |
| McVeigh, J.  2017  [**2,4-Dinitrophenol, the inferno drug: a netnographic study of user experiences in the quest for leanness.**](https://doi.org/10.3109/14659891.2016.1149238)  Public Health Institute, Liverpool John Moores University, Liverpool, UK. | Germain, J.  Van Hout, M. C. | **AIM: T**o explore the sharing of pharmacological knowledge, practice, and protocols for DNP use amongst individuals engaging in Internet forum activity.  **METHOD:** Netnography |  | - Communal folk pharmacological advice and recommendations for DNP manufacture and use with associated harms and outcomes were discussed. - The efficacy and negative effects of DNP were described and discussed. - DNP use was highly recommended for experienced bodybuilders only. - Descriptions of dosage and regimes for optimal use were evident. | Continued pharmacovigilance and surveillance of DNP is warranted. | Establishing contact and maintaining communication and directly influencing the DNP community may be beneficial for effective harm reduction and health promotion strategy | Future research engaging with the online community is needed to reach sections of the population of users. | **⚫** |
| McVeigh, J.  2017  [**Anabolic steroids in the UK: an increasing issue for public health.**](https://doi.org/10.1080/09687637.2016.1245713)  Public Health Institute, Liverpool John Moores University, Liverpool, UK. | Begley, E. | **AIM: T**o identify changes in the extent and patterns of anabolic steroid use in the UK to better understand the public health implications.  **METHOD:** Retrospective data analysis of needle and syringe programme data and review of the evidence related to health harms. | Needle and syringe programme clients in Northwest of England. | - Dramatic increase in the number of AAS users accessing NSPs to become the main client group. - Increased mean age of steroid using clients from | With the identification of HIV in the population and signs of rising prevalence, public health cannot wait for successful preventive interventions to be established, necessitating the implementation of a robust harm reduction strategy. | There is a need for comprehensive interventions in the context of increasing numbers of AAS injectors.  Greater engagement efforts by outreach to gyms and other environments where AAS use is likely to be found are needed. | Future research to estimate the scale, patterns, and drivers of AAS use.  The nature of the illicit market and the position of the internet.  Research into most effective demand and harm reduction interventions. | **⚫**  **⚫**  ⚫ |
| Hope, V.  2017  [**Low levels of hepatitis C diagnosis and testing uptake among people who inject image and performance enhancing drugs in England and Wales, 2012-15.**](https://doi.org/10.1016/j.drugalcdep.2017.06.018)  Public Health Institute, Liverpool John Moores University, Liverpool, UK. | McVeigh, J.  Smith, J.  Glass, R.  Njoroge, J.  Tanner, C.  Parry, J.V.  Ncube, F.  Desai, M. | **AIM:** HCV testing uptake and undiagnosed infections are examined using data from a voluntary unlinked-anonymous survey.  **METHOD:** Retrospective data analysis | IPED injectors, England, and Wales | - One in 20 of those injecting IPEDs sampled had anti-HCV. - One in 70 of those injecting only IPEDs had antibodies. - Of those who injected IPEDs only, who had been infected with hepatitis C were not aware of this. - The minority who had injected both IPEDs and psychoactive drugs had a higher hepatitis C prevalence and higher uptake of testing than those who had just used IPEDs | The development, evaluation, and implementation of targeted interventions to improve the uptake of hepatitis C testing among people who inject IPEDs need to be is needed. | Targeted HCV testing interventions are required for those injecting IPEDs. | Further research is needed to improve uptake of BBV services | **⚫**  ⚫ |
| Harris, M.  2017  [**Intrasexual competition as a potential influence on anabolic-androgenic steroid use initiation.**](https://doi.org/10.1177/1359105317692145)  Department of Applied Psychology, Cardiff  Metropolitan University, Cardiff CF5 2YB, UK. | Dunn, M.  Alwyn, T. | **AIM: T**o examine the extent to which users and non-users of AAS differ in levels of intrasexual competition, to explore levels of intrasexual competition between AAS users with varying usage experience and to investigate how levels of intrasexual competition vary between non-AAS using bodybuilders with varying bodybuilding experience  **METHOD:** Survey | Male Bodybuilders | - A statistically significant difference in levels of intrasexual competition between users and non-users of AAS was found, whereby users displayed increased levels compared to non-users irrespective of training experience. - The difference was driven specifically by higher intrasexual competition in the novice group. - AAS users exhibit higher levels of intrasexual competition as they initiate bodybuilding. - Experienced users display lower levels of intrasexual competition than less experienced users. - Physical competitiveness at least would be predictably lower in older populations. | Not Discussed | Not Discussed | Future research is needed on the precise factors that initiate and perpetuate AAS use.  Research focusing on the usage of these life-threatening substances before the gap between the theory, problem and provision grows even further apart is warranted. | **⚫**  **⚫** |
| Hanley Santos, G.  2017  [**The risk environment of anabolic–androgenic steroid users in the UK: Examining motivations, practices and accounts of use.**](https://doi.org/10.1016/j.drugpo.2016.11.005)  School of Law, Criminology and Government, Plymouth University, Drake Circus, Plymouth PL4 8AA, UK. | Coomber, R. | **AIM:** To examine steroid user motivations, patterns of use, and the ways in which these practices are accounted for using a risk environment framework.  **METHOD:** Qualitative interviews | AAS users recruited at NSP. | - Steroid use patterns varied according to motivation for use, experience, and knowledge gained. - Most gained information from fellow users or suppliers. - Many users differentiated themselves from other groups of steroid users—for example, older users expressed concern over patterns of use of younger and (what they saw as) inexperienced steroid users. | More effective drug policies can be developed by examining the social contexts of steroid use and the social and cultural meanings individuals attach to their risk practices. | Harm reduction strategies need to be expanded.  Interventions for steroid users need to understand the heterogeneity of users and the specific local and wider contexts of their use.  School/college-based, community- based and community-level interventions with appropriate peer and social network involvement that address issues around body image, stigma and misinformation are needed. | Research examining social contexts of use and user beliefs and motivations is vital to understanding how ‘risk’ behaviours are experienced.  Further research is needed into effective alternative drug policies, such as regulation. | **⚫** |
| Hall, A.  2017  [**Illicit pharmaceutical networks in Europe: organising the illicit medicine market in the United Kingdom and the Netherlands.**](https://link.springer.com/article/10.1007/s12117-017-9304-9)  School of Social Sciences, Business and Law, Teesside University, Middlesbrough, UK. | Koenraadt, R.  Antonopoulos, G. A. | **AIM:**  **METHOD: Analysis of both on** Data analysis from two research projects including online and traditional methodologies. |  | *Study 1 UK:*   - to explore and examine the size, scope, nature, and dynamics of the illicit medicine market in the UK, focusing on the demand and supply. - qualitative methods both online and offline settings   *Study 2 Netherlands:*   - focused on the supply and demand processes associated with the trade in illicit medicines in the Netherlands. - multi-method approach   *Main Findings of the current study:*   - The market is facilitated by numerous small groups with varying coherence, and in the case of largescale smuggling, networks. - The research has also identified business relationships generated and sustained online. - The UK serves as a transit country and the Netherlands, as a transit country, is also increasingly used as a manufacturing hub. | Not Discussed | Not Discussed | Not Discussed | **⚫** |
| Chatwin, C.  2017  [**New drugs, new directions? Research priorities for new psychoactive substances and human enhancement drugs.**](https://doi.org/10.1016/j.drugpo.2017.01.016)  University of Kent, United Kingdom. | Measham, F.  O'Brien, K.  Sumnall, H. | **METHOD:** Editorial |  | - Information about new drugs in general, both new psychoactive substances and human enhancement drugs is urgently needed. - An improved evidence base for policymakers and practitioners to draw upon will promote meaningful progress in responding to the new drug phenomenon. | Assessment and evaluation of the new legislative landscapes that are developing as a direct result of rising anxieties about new drugs, often based around precautionary principles rather than strong evidence of harm and the need for intervention is warranted. | Not Discussed | Research on new drugs should pay more attention to the intersections between traditional drugs, NPS and HED.  Research exploring new drug use amongst vulnerable populations such as the prison population and those who have recently been released from prison, the homeless, and those who are experiencing mental health problems is urgently required. | **⚫**  ⚫  **⚫** |
| Brennan, R.  2017  [**The injecting use of image and performance-enhancing drugs (IPED) in the general population: a systematic review.**](https://doi.org/10.1111/hsc.12326)  School of Health Science, Waterford Institute of Technology, Cork Road Waterford, Ireland.  **Co-authors: UK** | Wells, J.S.G.  Van Hout, M.C. | **AIM:** To review the injecting use of image and performance-enhancing drugs (IPED) in the general population.  **METHOD:** Systematic review | IPED injectors | - Motivations for the use of IPEDs are grounded in appearance, pursuit of health and youth, and body image disturbance. - Moderated use was reported. - Pathological use was associated with high-risk behaviours, which may be normalised in IPED communities. - Insufficient scientific reporting of IPED trajectories and pathways of use. - IPED specific discussion forums are an underutilised resource for identifying IPED trajectories and pathways of use. | Updated publication information is necessary for dissemination to health and social policy makers and those in healthcare practice. | Community-based clinical practice and health promotion services need to consider this review which highlights the patterns of this public health issue. | Future online research methods are warranted to investigate prevalence and patterns of injecting use, and to map health outcomes in IPED users. | **⚫**  **⚫**  **⚫** |
| Boardley, I.  2017  [**Empathic and Self-Regulatory Processes Governing Doping Behavior.**](https://dx.doi.org/10.3389%2Ffpsyg.2017.01495)  School of Sport, Exercise & Rehabilitation Sciences, University of Birmingham, Birmingham, UK | Smith, A. L.  Mills, J. P.  Grix, J.  Wynne, C. | **AIM:** To test a model of doping behaviour with team- and individual-sport athletes and corporate- and hardcore-gym exercisers  **METHOD:** Questionnaire | Athletes and hardcore gym users | - The efficacy of a model based on Bandura’s (1991) theory was supported using structural equation modelling. - Empathy and doping SRE negatively predicted reported doping. - The predictive effects of empathy and doping SRE on reported doping were mediated by doping MD and anticipated guilt. - Doping MD positively predicted reported doping. - The predictive effects of doping MD on reported doping were partially mediated by anticipated guilt. - The model-testing aspects of the project were based on cross-sectional data; the causal nature of the predictive effects identified could not be tested and therefore should not be inferred. | Not Discussed | Not Discussed | Research that builds on this work by employing experimental or quasi-experimental designs to test the causal nature of the identified associations is recommended.  Longitudinal research testing the temporal ordering proposed in the model tested is encouraged.  Future researchers should explore alternative approaches to the assessment of doping susceptibility. | **⚫**  **⚫** |
| Begley, E.  2017  [**Image and Performance Enhancing Drugs: 2016 National Survey Results.**](https://www.wales.nhs.uk/sitesplus/documents/888/IPED%20report%202017.%20FINAL.pdf)  Public Health Institute, Liverpool John Moores University, Liverpool, UK. | McVeigh, J.  Hope, V. D.  Bates, G.  Glass, R.  Campbell, J.  Tanner, C.  Kean, J.  Morgan, G.  Acreman, D.  Smith, J. | **AIM:** To explore image and performance drug use in Wales, England, and Scotland.  **METHOD:** Survey at gyms and NSPs. | IPED Users | - AAS most reported IPED used – - 89% ever used oral IPEDs, 85% ever injected. - More than half would not seek medical advice for an adverse effect of use. - 74% reported psychoactive drug use. | Informing policy and practice is challenging as people using IPEDs report the use of a broad range of other illicit drugs (cannabis, cocaine, and ecstasy) and/or over the counter pain relief medication. | Targeted interventions to address the sexual health needs of IPED users is required. | Further research is warranted to investigate and understand the combinations of drugs used and drug use practices among people using IPEDs.  Effective and targeted qualitative research is needed to explore the reasons for low uptake of testing for blood borne viruses and hepatitis B vaccination. | **⚫**  **⚫**  **⚫**  ⚫  **⚫** |
| Bates, G.  2017  [**HIV among people using anabolic steroids in the United Kingdom: an overview.**](https://hivnursing.net/wp-content/uploads/2020/11/HIV_Nursing_17_1_20.pdf)  Public Health Institute, Liverpool John Moores University, Liverpool, UK. | Hope, V. D.  McVeigh, J. | **AIM:** To discuss anabolic steroid use in the context of HIV  **METHOD:** Review |  | - Increasing awareness and an understanding of the characteristics and risks of people who use AS to support healthcare professionals is needed. - HIV testing should be offered to people using AAS presenting at healthcare services for blood tests or physiological or psychological treatment, but also if AAS use is suspected during routine healthcare appointments. - It is essential that nurses and other healthcare professionals capitalise on engagement opportunities while avoiding stigma and the potential for further barriers to engagement. | Not Discussed | Health professionals aim to reduce risk behaviours and prevent the spread of infections among all people who use drugs including those who use AAS is challenging.  Healthcare services must find opportunities to offer testing and treatment for BBV, as well as advice and information regarding injecting behaviours and sexual health.  Nurses and other healthcare professionals can apply their skills and knowledge to deliver interventions to people using AAS, including HIV testing, and increasing their awareness of AAS use and associated risks will support this. | Further research into the transmission of BBV among those who use AAS is required.  Further research is required to better understand facilitators and barriers to the use of appropriate healthcare services by this population | **⚫**  ⚫ |
| Van Hout, M.C.  2016  [**Netnography of Female Use of the Synthetic Growth Hormone CJC-1295: Pulses and Potions.**](https://doi.org/10.3109/10826084.2015.1082595)  Waterford Institute of Technology, Health, Sport and Exercise Science, Waterford, Ireland.  **Co-authors: UK** | Hearne, E. | **AIM:** To explore female use of CJC-1295, a synthetic growth hormone analogue from the perspectives contained in Internet forum activity.  **METHOD:** Netnography | IPED Users in online fora | - Forum users were well versed and experienced in the poly use of IPEDs. - Choice to use CJC-1295 centred on weight loss, muscle enhancement, youthful skin, improved sleep, and injury healing. - Concerns were described relating to female consequences of use given gender variations in growth hormone pulses affecting estimation of dosage, cycling, and long-term consequences. | Not Discussed | Public health interventions should consider female self-medicating use of synthetic growth hormone within a repertoire of product supplementation, and related adverse health consequences.  Credible forms of health education tactics should accurately advise users of health risks, hazards associated with counterfeit products and illicit sourcing of these supplements. | Further research is warranted into the neurobiology of growth hormone abuse to investigate long-term effects and inform health professionals and users.  Continued development of Internet methodologies. | **⚫** |
| Smith, D.  2016  [**Muscle Dysmorphia and Anabolic-Androgenic Steroid Use.**](https://link.springer.com/chapter/10.1057/978-1-137-53535-1_3)  MMU, Exercise and Sport Science, Manchester, UK | Rutty, M.C.  Olrich, T.W. | METHOD: Review |  | - The authors argue that body image dissatisfaction should be a concern to all those interested in mental health. - Simple messages that the use of AAS can damage long-term health are unlikely on their own to be effective in this population. | Not Discussed | Care must be taken with those who have ceased AAS use to encourage them to develop other interests that may at least reduce their reliance on their body as their sole source of self-esteem.  Caution should be exercised with ant-depressant prescribing particularly combined with the low testosterone levels that result from cycling off AAS, suicidal ideation can result in some individuals. | Large scale research to explore the characteristics of MD with former AAS users to see if they self-identify times in their lives when they behaved in such ways and suffered from MD would be beneficial.  An exploration of how individuals have attempted to overcome feelings of MD is warranted. | ⚫  **⚫** |
| McVeigh, J.  2016  [**Harm reduction interventions should encompass people who inject image and performance enhancing drugs.**](https://doi.org/10.1136/bmj.i1889)  Public Health Institute, Liverpool John Moores University, Liverpool, UK | Kimergard, A.  Bates, G.  Hope, V. D. | **METHOD:** Letter | N/A | - The authors support the role of harm reduction in the “fight” against HIV,1. - The authors broadly agree with the findings of the report “The Case for a Harm Reduction Decade: Progress, Potential and Paradigm Shifts”. - IPED users are a growing client group in many countries with longstanding provision of needle and syringe programmes. - HIV prevalence of 1.5% in men who inject IPEDs reported. | The use of IPEDs, particularly the injection of AAS by men, must be viewed as a serious public health concern requiring the attention of policy makers. | Harm reduction interventions should address the needs of emerging and often hidden populations of people who inject drugs. | Consistent research in relation to emerging patterns of injecting drug use is warranted. | **⚫**  ⚫ |
| Joubert, E.  2016  [**Considering anabolic androgenic steroid use in relation to non-substance related diagnostic categories with special emphasis on eating disorders: a systematic review.**](https://doi.org/10.3109/14659891.2014.977974)  Department of Psychosexual Service, South London and Maudsley NHS Foundation Trust, Maudsley Hospital, London, UK | Melluish, S. | **AIM:** To conduct a systematic review of current literature considering how best to diagnose and conceptualise AAS.  **METHOD:** Systematic review |  | - A traditional understanding of AAS use as substance abuse was preferred by some. - Numerous indicators support an understanding which places AAS use within a larger disorder such as OCD or an eating disorder. - The largest body of literature to date suggests placing AAS use within either the category of an eating disorder or BDD. - Many similarities between the psychological profile of AAS users (mostly male) and anorexic patients (mostly female. - A major finding from this review is that the number of steroid users taking part in the included studies is very low compared to the number of users. |  |  | Future research to understand ASS use is recommended to influences the provision of appropriate treatment.  Future research should consider novel and alternative ways to recruit participants to have a larger number from more diverse backgrounds in future studies | **⚫**  **⚫** |
| Iversen, J.  2016  [**Access to needle and syringe programs by people who inject image and performance enhancing drugs.**](https://doi.org/10.1016/j.drugpo.2016.01.016)  Public Health Institute, Liverpool John Moores University, Liverpool, UK. | Hope, V. D.  McVeigh, J. | **METHOD:** Commentary |  | - The authors appreciate van Beek and Chronister’s concerns regarding the funding of harm reduction interventions in an environment of diminishing resources (van Beek & Chronister, 2015). - The authors disagree with their conclusion and support the international guidelines for equitable and non-discriminatory NSP provision for all people who inject drugs. | Consideration of the potential for all types of injecting drug use to increase the risk of transmission of BBVs and cause other harms is warranted. | A screening process to identify ‘need’ of specific groups of people who inject drugs, in which those injecting IPEDs are deemed to be at negligible risk of BBV transmission, may result in increased complacency and have a negative impact for practice. |  | **⚫**  **⚫**  **⚫** |
| Hope, V.  2016  [**Risk of HIV and Hepatitis B and C Over Time Among Men Who Inject Image and Performance Enhancing Drugs in England and Wales: Results from Cross-Sectional Prevalence Surveys, 1992-2013.**](https://doi.org/10.1097/qai.0000000000000835)  Public Health Institute, Liverpool John Moores University, Liverpool, UK. | Harris, R.  McVeigh, J.  Cullen, K. J.  Smith, J.  Parry, J. V.  DeAngelis, D.  Ncube, F. | **AIM:** Blood-borne virus prevalence trends among IPED injectors are explored.  **METHOD:** Retrospective data analysis. |  | - BBV infections are a problem among people who inject IPEDs. - There is an overlap of BBV infections with the MSM population. - BBV prevalence among IPED injectors has increased. - HIV prevalence is now like that among psychoactive drug injectors. - Both sexual and injection risks lead to BBV transmission among those injecting IPEDs. |  | Targeted interventions to reduce risks are indicated.  There is a need for targeted interventions to address sexual health and drug use risks among those injecting IPEDs. | The role of targeted interventions to address how sexual health and drug use risks among those injecting IPEDs, factors in the changing BBV prevalence requires further investigation. | **⚫** |
| Harris, M.A.  2016  [**A qualitative exploration of the motivations underlying anabolic-androgenic steroid use from adolescence into adulthood.**](https://psycnet.apa.org/doi/10.5114/hpr.2016.61669)  Cardiff Metropolitan University, United Kingdom. | Dunn, M.  Alwyn, T. | **AIM:** To explore the direct experience of anabolic androgenic steroid (AAS) use by young men, with an emphasis on how motivations progressed from adolescent initiation to more entrenched usage  **METHOD:** Qualitative interviews | Male AAS users | - Body image pathology and problem behaviours highly associated with AAS use was identified. - A transitional period whereby AAS use, and bodybuilding fuelled the expression of a body image problem, rather than a body image problem precipitating AAS use was identified. - Intense pressure on gaining muscularity manifests itself into a problematic pattern of body image concerns. - A pattern of problem behaviours to be highly prominent during the initiation of AAS use was revealed. |  | A more complex relationship between body image and AAS use than previously suggested requires attention by those in practice and healthcare. | Future research into the extent to which body image problems initiate or perpetuate the use of AAS warrants further examination. | **⚫**  **⚫** |
| Hall, M.  2016  [**Bodybuilders' accounts of synthol use: The construction of lay expertise online.**](https://doi.org/10.1177%2F1359105314568579)  Lancaster University, UK. | Grogan, S.  Gough, B. |  | Male bodybuilders who discuss their Synthol Use (*n=*17) | - Study provided insights into how these users positioned themselves as novices and experts in this discussion group. - Users clearly expect that synthol will give them a natural, trained look and are keen for advice on how to use synthol and get good results while minimising unwanted side effects such as pain and unsightly bumps. - People with practical experience of use are treated with respect and trusted to provide technical and medical information which is factual and generalisable beyond personal experience of use. |  |  | Medically accurate information that has credibility with bodybuilders to enable ‘novice’ users to have somewhere to go to find accurate information on health risks and safer use is needed. | **⚫** |
| Hall, A.  2016  [**Fake Meds Online the Internet and the Transnational Market in Illicit Pharmaceuticals.**](https://www.palgrave.com/gp/book/9781137570871)  Department of Social Sciences at Northumbria University. | Antonopoulos, Georgios A | **AIM:** To provide an in-depth and empirically grounded social scientific analysis of the online trade in illicit medicinal products.  **METHOD:** Virtual and traditional ethnography; qualitative interviews; secondary data. |  | - This book provides a timely criminological investigation into the sale of fake medicines online. Chapters focus on:   1. The Online Trade in Illicit Pharmaceuticals: The UK Context  2. The Demand Side  3. The Supply Side | Policymakers need to consider that the supply of illicit medicines is embedded in political forces, economic processes and technological advancements that blur the boundaries of legality. |  | Research is needed to understand further the serious side effects of substances discussed in this book when taken inappropriately or in high doses, and the risk of purchasing online. | **⚫** |
| Graham, M.R.  2016  [**Peptide Hormones, Metformin and New-Wave Practices and Research Therapies.**](https://link.springer.com/chapter/10.1057/978-1-137-53535-1_11)  School of Science & Sport, Llantarnam Research Academy, Cwmbran, UK | Baker, J. S.  Davies, B. | **METHOD:** Review |  | All drugs or medicines have side effects, which are exacerbated if the dosages taken are excessive.  In the UK, the Internet has allowed the general population to self-medicate by importation of medicines that were previously otherwise unavailable. | Policy consideration is needed with respect to UK legislation |  | More research is needed to explore the country specific legislation issues. | **⚫**  **⚫** |
| Edwards, C.  2016  [**Predicting muscularity-related behaviour, emotions, and cognitions in men: The role of psychological need thwarting, drive for muscularity, and mesomorphic internalization.**](https://doi.org/10.1016/j.bodyim.2016.06.005)  Institute of Sports & Exercise Science, University of Worcester, Worcester, UK. | Tod, D.  Molnar, G.  Markland, D. | **AIM:** To examine the relationships of internalization, need thwarting (NT), and drive for muscularity (DFM) – and their interactions – with self-reported weightlifting, muscle dysmorphia (MD) and muscle-related worry (MRW).  **METHOD:** Questionnaire | 552 men | - DFM significantly predicted weightlifting, MRW, and MD. - Internalization significantly predicted weightlifting and MRW. - NT significantly predicted weightlifting and MD. - The interactions between NT and DFM and NT and internalization predicted MD. | Not discussed | When muscularity-focused issues are tied to basic psychological needs, some assistance or prevention strategies based on simple relationships between internalization, DFM, and outcomes may not be effective. | Future research to explore the role of NT, DFM, and internalization in predicting other appearance-focussed behaviours in men (e.g., disordered eating, steroid use) is needed. | **⚫** |
| Coxon, J.  2016  [**Hypogonadism persistence after long-term Illicit use of anabolic-androgenic steroids.**](https://doi.org/10.1016/j.jsxm.2016.03.238)  Brighton, United Kingdom. |  | **AIM:** N/A  **METHOD:** Clinical case report | Male, 38 years | - A 38-year-old man presented with erectile dysfunction and low energy. - History of high-dose AAS use for 7-8 years; ceased use 3 years previously. - Test revealed mildly raised BMI of 28. - Low total testosterone. - MRI scan revealed no abnormalities. - Prescribed clomiphene citrate, 50mg on alternate days. - 3 months later, total, and free testosterone levels had increased a little. - May indicate that duration of the hypogonadal symptoms greater in those who use higher doses, and for a longer time, and older men. |  |  | Future research should focus on exploring previous use of AAS. | **⚫**  **⚫** |
| Boardley, I.  2016  [**Nutritional, medicinal, and performance enhancing supplementation in dance.**](https://doi.org/10.1016/j.peh.2015.11.005)  School of Sport, Exercise & Rehabilitation Sciences, University of Birmingham, Birmingham, UK. | Allen, N.  Simmons, A.  Laws, H. | **AIM:** To investigate the reported prevalence of nutritional, medicinal, and performance enhancing substance use in dance; and to examine the amount of importance dancers place on potential sources of information regarding supplementation.  **METHOD:** Survey | Male and female UK-based dancers (371) | - 91.9% of the dancers used at least one supplement. - Highest prevalence rates were for multivitamins, over-the-counter painkillers, and high-energy drinks. - Prevalence of PED use was low. - The most important sources for supplementation information were physiotherapists and GP/physicians. | If research supports a need to control the use of some substances in dance, attention must be given on how to encourage safe supplementation considering the issues with anti-doping policy in sport. | Consideration for dancers and how they can be supported optimally to protect their health is warranted. | Replication of the current study using alternative approaches such as the Randomised Response Technique (RRT) is recommended for future research.  An investigation to identify if the types of supplements used, frequency and volume of use, change across the performance season may be beneficial. | **⚫**  **⚫**  **⚫** |
| Bates, G.  2016  [**Image and Performance Enhancing Drugs 2015 Survey Results.**](https://www.ljmu.ac.uk/~/media/phi-reports/pdf/2016_07_image_and_performance_enhancing_drugs_2015_survey_results.pdf)  Public Health Institute, Liverpool John Moores University, Liverpool, England. | McVeigh, J. | **AIM:** To explore image and performance drug use in Wales, England, and Scotland.  **METHOD:** Survey predominantly at NSPs | IPED Users | - Study focused on all IPEDs. Majority recruited at NSPs or outreach settings. - Complex polypharmacy reported including alcohol and psychoactive drug use. - Reasons to use were primarily for muscle gain followed improving strength and fat loss. - IPED using populations, practices and health risks are ever changing with a dynamic fast-paced market. |  | The results indicate that understanding how to effectively engage IPED users with health services for the treatment of adverse health effects associated with their IPED use, as well as identifying successful ways to improve blood borne virus testing in this population, is critical. | Future research is recommended to routinely investigate IPED drug practices and related behaviours, and health outcomes.  An in-depth exploration of IPED sub-groups is warranted for the establishment of prevention, harm reduction and treatment interventions. | **⚫**  **⚫**  **⚫**  ⚫  **⚫** |
| Antonopoulos, G. A.  2016  [**"Gain with no pain': Anabolic-androgenic steroids trafficking in the UK.**](https://doi.org/10.1177%2F1477370816633261)  Teesside University, UK. | Hall, A. | **AIM:** To provide an account of the social organization of the steroids trafficking business in the UK.  **METHOD:** Review. |  | - The steroid trade is diverse, adaptable and with no hierarchies. - The steroids market encompasses a multitude of individuals who flow between legality and illegality, online and offline, and for both use and supply. |  |  | In-depth understanding of the complexities of the illicit anabolic steroid trade is recommended for future researchers. | **⚫** |
